# Codon optimization with deep learning to enhance protein expression

**DOI:** 10.1038/s41598-020-74091-z

**Published:** 2020-10-19

**Authors:** Hongguang Fu, Yanbing Liang, Xiuqin Zhong, ZhiLing Pan, Lei Huang, HaiLin Zhang, Yang Xu, Wei Zhou, Zhong Liu

**Affiliations:** 1 University of Electronic Science and Technology of China, Chengdu, 611731 China; 2 State Key Laboratory of Biotherapy, West China Hospital, Sichuan University, Chengdu, 610041 China; 3 Chengdu Institute of Computer Applications, Chinese Academy of Sciences, Chengdu, 610041 China

**Keywords:** Bioinformatics, Gene expression analysis, Biotechnology

## Abstract

Heterologous expression is the main approach for recombinant protein production ingenetic synthesis, for which codon optimization is necessary. The existing optimization methods are based on biological indexes. In this paper, we propose a novel codon optimization method based on deep learning. First, we introduce the concept of codon boxes, via which DNA sequences can be recoded into codon box sequences while ignoring the order of bases. Then, the problem of codon optimization can be converted to sequence annotation of corresponding amino acids with codon boxes. The codon optimization models for *Escherichia Coli* were trained by the Bidirectional Long-Short-Term Memory Conditional Random Field. Theoretically, deep learning is a good method to obtain the distribution characteristics of DNA. In addition to the comparison of the codon adaptation index, protein expression experiments for *plasmodium falciparum* candidate vaccine and polymerase acidic protein were implemented for comparison with the original sequences and the optimized sequences from Genewiz and ThermoFisher. The results show that our method for enhancing protein expression is efficient and competitive.

## Introduction

With the rapid development of biotechnology, heterologous expression has been utilized to generate recombinant proteins for use in vaccines and pharmaceuticals^1,2^. The codon is the basic unit of correspondence between nucleic acids carrying information and proteins carrying information and is also the basic link for information transfer in vivo. Codons that encode the same amino acid are called synonymous codons. While the usage probabilities of synonymous codons are not the same during protein synthesis, a species or a gene typically prefers to use one or several specific synonymous codons called optimal codons, and this phenomenon is known as codon usage bias^3^. Moreover, the codon usage bias of genes differs significantly among different functions.

Codon usage bias has a complex effect on protein expression levels when recombinant proteins are heterologously expressed^4^. The frequency of codons in a DNA sequence is positively correlated with the corresponding tRNA in a species, and the tRNA concentration determines the number of amino acids available for protein translation extension, which in turn affects the efficiency of protein synthesis^5,6^. Thus, the expression levels of proteins are highly correlated with codon usage bias. Rare codons tend to reduce the rate of translation and even cause translation errors^7^. Furthermore, codon optimization is the most critical determinant of increasing protein expression^8^. In gene synthesis, codon optimization involves recombination based on different criteria without changing the sequence of the amino acid^9^ and can promote expression of the recombinant gene in different host organisms^9–11^. Therefore, codon optimization for microorganisms is an essential part of gene synthesis.

In heterologous expression systems, to maximize protein expression from the DNA sequence of the original species in the host, codon optimization improves the translation efficiency of a target gene^12^ by converting the DNA sequence of nucleotides of one species to that of another, such as converting human sequences to bacterial or yeast sequences, plant sequences to human sequences, and fungal sequences to yeast sequences. Various codon optimization strategies have been developed by using a range of quantitative methods to generate different mRNA sequences, which can result in different levels of final protein expression. Most optimization strategies use codons with host bias to replace less frequently occurring codons^13–16^. In addition, a strategy is proposed to adjust the original codon sequence to match the natural distribution of the host codons^13,17–19^, the goal of which is to preserve the slow translation regions that are important for protein folding^9,10,20^. This strategy has been recognized as the best way to optimize codons.

In the industry, many biotechnology companies perform codon optimization, such as ThermoFisher (www.thermofisher.com) and Genewiz (www.genewiz.com), whose methods are based on the aforementioned strategies and empirical indexes. As a consequence, their indexes for codon optimization mainly include the codon adaptation index (CAI)^21^, the frequency of relative synonymous codon usage^22^, the codon bias index^23^, optimal codon usage^7^, and effective codon number^24^. Among these indexes, the CAI is the primary index used to predict gene expression level because it indicates the extent to which the coding sequence represents the usage of codons in an organism^25^.

In addition to the strategies considered to eliminate rare codons, there also exist parameters with important impacts on protein expression, such as the GC content^26^, RNA secondary structure, cleavage sites, restriction endonuclease sites, repeats and certain added or deleted motifs^27,28^. Many websites and software incorporate codon optimization algorithms with various determinants, such as DNA Works^29^, Optimizer^30^, GeMS^31^, Gene Designer^14^, Gene Designer Synthetic^32^, ThermoFisher and Genewiz. To further optimize the DNA sequence, some researchers also perform plasmid-mediated replenishment of tRNAs corresponding to rare codons from the host^33^. Currently, emerging high-throughput methods for gene synthesis and screening can also increase protein expression levels^34^.

In fact, the method that directly replaces rare codons with host biased codons is straightforward and can be implemented easily. However, the optimized DNA sequences contain host biased codons, so the transcribed mRNA contains a high percentage of codon subsets, which results in an imbalance of different tRNAs and eventually leads to the depletion of tRNA and termination of translation^14^. Additionally, the method that coordinates and replaces codons can make the adjusted codon sequence consistent with the natural distribution in the host, but this method is often complicated to implement and lacks flexibility.

The CAI is an important index with which to measure protein expression, but it is not comprehensive. Therefore, we use the deep learning method instead of the index method. The CAI is used only as a reference index in this paper, and it is verified by biological experiments.

Recently, deep learning has shown impressive applicability in a variety of domains, entailing a series of machine learning algorithms. Biological and medical research is replete with big data, but the data are often perplexing. These problems might be more appropriately handled using deep learning techniques^35^. The original idea stems from applying deep learning techniques to obtain the distribution of codons for feasible codon optimization without any empirical rules.

In this study, the concept of a codon box is introduced as a method to recode DNA sequences. Next, a popular sequence annotation method in deep learning called Bidirectional Long-Short-Term Memory Conditional Random Field (BiLSTM-CRF)^36^ was adopted to annotate amino acid sequences with codon boxes or codons directly. Finally, biological experiments were conducted to analyze and compare the protein expression in *Escherichia coli* (*E. coli*) with that obtained by Genewiz and ThermoFisher.

## Results

### Codon box

Regardless of the base order of the codons, or equivalently, if the codons contain the same A, T, G, and C bases, then they are taken as a set, which is called a codon box. For example, the codons ATG, TAG, AGT and GAT are taken as a whole codon box {agt}, as shown in Table 1. It is coincidental that the total number ofcodon boxes is 20, which is exactly equal to the number of amino acids in the universal codon table.

**Table 1:** Classification of codon boxes.

Type of codon box	Codon box	Amino acid	Codon
Type-1	{aaa}	Lys	AAA
	{ccc}	Pro	CCC
	{ggg}	Gly	GGG
	{ttt}	Phe	TTT
Type-2	{aac}	Gln, Asn, Thr	CAA, AAC, ACA
	{aag}	Arg, Glu, Lys	AGA, GAA, AAG
	{aat}	Ile, Asn	ATA, AAT
	{acc}	His, Pro, Thr	CAC, CCA, ACC
	{agg}	Arg, Glu, Gly	AGG, GAG, GGA
	{att}	Ile, Leu, Tyr	ATT, TTA, TAT
	{ccg}	Ala, Arg, Pro	GCC, CGC, CCG
	{cct}	Leu, Pro, Ser	CTC, CCT, TCC
	{cgg}	Ala, Arg, Gly	GCG, CGG, GGC
	{ctt}	Leu, Phe, Ser	CTT, TTC, TCT
	{ggt}	Gly, Trp, Val	GGT, TGG, GTG
	{gtt}	Cys, Leu, Val	TGT, TTG, GTT
Type-3	{acg}	Ala, Arg, Asp, Gln, Ser, Thr	GCA, CGA, GAC, CAG, AGC, ACG
	{act}	His, Ile, Leu, Ser, Thr, Tyr	CAT, ATC, CTA, TCA, ACT, TAC
	{agt}	Asp, Met, Ser, Val	GAT, ATG, AGT, GTA
	{cgt}	Ala, Arg, Cys, Leu, Ser, Val	GCT, CGT, TGC, CTG, TCG, GTC

According to the codon box concept, 64 codons can be divided into 20 kinds of codon boxes. Furthermore, the codon boxes can be classified into three categories: Type-1 has only one kind of base; Type-2 has two kinds of bases; and Type-3 has three kinds of bases.

**Figure 1:**
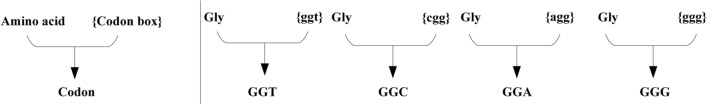
One-to-one mapping of amino acids and codon boxes with codons. An example of how an amino acid (Gly and its corresponding codon box can uniquely determine a codon.

Table 1 also shows that the codon encoding a given amino acid can be uniquely determined by a codon box and the amino acid, that is, different codons in the same codon box cannot encode the same amino acid. For example, the codons encoding the amino acid Gly are GGT, GGC, GGA and GGG. The codon box {ggt} contains GGT, TGG and GTG. Therefore, once the amino acid Gly and the codon box {ggt} are given, GGT can be uniquely determined to encode Gly, as shown in Fig. 1. For other codon boxes, the corresponding codons encoding Gly are determined similarly. This critical property has not been identified previously and can play a key role in subsequent codon optimization.The codon box can be regarded as a coding method in machine learning that can simplify deep learning models, and a codon box and an amino acid together can uniquely determine a codon, which has not been reported previously. Furthermore, it was verified that the effect is better after introducing a codon box. The number of codon boxes was consistent with that of conventional amino acids. However, whether the use of codon boxes is directly relevant in biology needs further study.

### Codon optimization with deep learning

**Figure 2:**
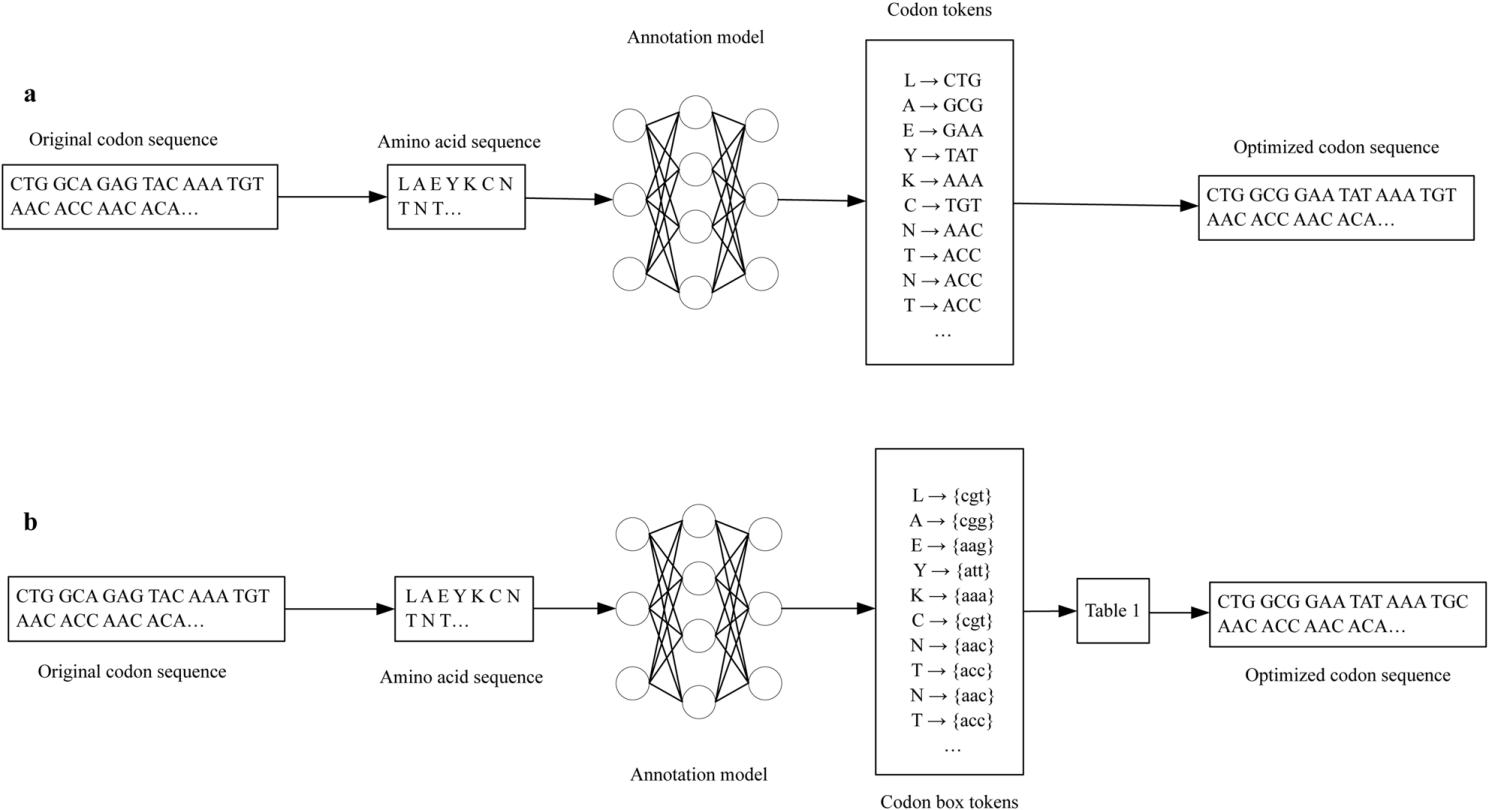
Codon optimization flowcharts based on sequence annotation models. First, the original codon sequences are decoded into amino acid sequences. Then, they are annotated by the trained sequence annotation models. In the flowchart in (**a**), the amino acid sequence is annotated with 61 kinds of codons, except stop codons (named BiLSTM-CRF(a)), and in the flowchart in (**b**), the amino acid sequence is annotated with 20 kinds of codon boxes (named BiLSTM-CRF(b)). The difference in (**b**) is that the optimized codons are determined from the codon boxes in Table 1 due to the one-to-one mapping of amino acids and codon boxes with codons mentioned in the previous section. Generally, the annotation model with fewer tokens is better, and the complexity of BiLSTM-CRF(b) is lower than that of BiLSTM-CRF(a).

The choices of synonymous codon pairs are not random in individuals^3^, and different species are subject to different rules embedded in the distribution of their codons. To accurately capture the codon distribution of host genes, the codon optimization problem can be converted to that of a sequence annotation problem in deep learning, as shown in Fig. 2. BiLSTM-CRF is the most widely used sequence annotation algorithm, and the code for the BiLSTM-CRF annotation method is available at https://github.com/jiesutd/NCRFpp. In this paper, our focus is not the algorithm design for BiLSTM-CRF, as shown in Fig. 2a, which is simply a training tool for the *E. coli* codon optimization model. Our method, based on the codon box in Table 1, is available at https://github.com/Devil625/Codon_Optimization.git, whose flowchart is shown in Fig. 2b.

It is obvious that the outputs of the two kinds of annotation models were designed according to the codon distribution of host genes. To train the annotation models, the training data including 4906 genes were selected from the DNA sequences of *E. coli* available from the NCBI, 80% of which were used as the training set, 10% as the validation set, and 10% as the test set. In the models, every amino acid is considered to be a word, and the dimension of the word vector is an important parameter. Considering 20 kinds of amino acids and stop codons, a word vector with 21 dimensions is a proper selection for word-embedding vectors of amino acid sequences. In practical training, the CAI indicates that a word vector with 21 dimensions can yield a better result than one with 50 dimensions or 100 dimensions.

Because gene mutation cannot theoretically be avoided in sequence annotation, that is, the optimized codon sequence may encode a different amino acid, the model with fewer mutations is better. In the case of a mutation, the mutant codon will be replaced with the original codon to ensure that the amino acid sequence remains unchanged. Surprisingly, there was almost no mutation in the training process.

Machine learning approach is a probabilistic model and therefore cannot rule out the possibility of mutation. To date, we have performed many experiments with data from the related protein expression optimization literature, and have not found such mutation. To further study the probability of such mutation, we randomly generated 10,000 genes and found that the mutation probability was 0.00%. Therefore, this operation did not alter the effect of our model.

The other hyperparameters also have significant impacts on performance when training the BiLSTM-CRF model. In this paper, the main hyperparameters of our model were selected as follows: a 4-layer BiLSTM was selected, and the hidden layer dimension was 200; the dropout was 0.5; the batch size was 32; and the learning rate was 0.003. To verify the rationality of the codon box proposed in this paper, BiLSTM-CRF(a) and BiLSTM-CRF(b) were trained in the same environment. The training times for BiLSTM-CRF(a) and BiLSTM-CRF(b) were approximately 40 h and 17 h on 1080 GPU, respectively; the test accuracy and training accuracy of BiLSTM-CRF(a) were 0.52 and 0.76, respectively; and the test accuracy and training accuracy of BiLSTM-CRF(b) were 0.52 and 0.77, respectively. BiLSTM-CRF(a) and BiLSTM-CRF(b) have almost the same model accuracy.

Because our goal in this paper was the optimization of DNA sequences, CAI is used as a main index of model comparison. The CAI is an important index with which to measure protein expression, but it is not comprehensive. Therefore, we use the deep learning method instead of the index method. The CAI is used only as a reference index in this paper, and it is verified by biological experiments. The average CAIs of BiLSTM-CRF(a) and BiLSTM-CRF(b) for the test set were 0.94 and 0.96, respectively. According to the statement on GeneScript's optimization website^37^, the ideal range for CAI is 0.8–1.0, and the lower the number is, the higher the chance that the gene will be expressed poorly. Therefore, BiLSTM-CRF(b) is better than BiLSTM-CRF(a) in terms of training time and the CAI.

To compare the CAI of the original sequence with those of the Genewiz, ThermoFisher, BiLSTM-CRF(a), and BiLSTM-CRF(b) optimized sequences, six codon sequences (HPDF, PAE, MMPL3, FALVAC-1, PA and PTP4A3) were extracted from six papers on gene optimization and protein expression1^38–42^, as shown in Table 2, from which the 972 bp plasmodium falciparum candidate vaccine (FALVAC-1) and 564 bp polymerase acidic protein (PTP4A3) were randomly selected for biological experiments on protein expression.

**Table 2:** CAI comparison between original sequences and optimized sequences.

DNA	bp	Original	Genewiz	ThermoFisher	BiLSTM-CRF(a)	BiLSTM-CRF(b)
HPDF	615	0.70	0.85	0.92	0.96	0.98
PAE	1839	0.76	0.81	0.92	0.96	0.98
MMPL3	2835	0.67	0.79	0.93	0.96	0.98
FALVAC-1	972	0.67	0.85	0.93	0.95	0.96
PA	561	0.60	0.83	0.93	0.97	0.98
PTP4A3	564	0.70	0.83	0.93	0.96	0.98
Average	1231	0.68	0.83	0.93	0.96	0.98

This table shows the CAIs of the sequences optimized by different optimization tools, among which the values for Genewiz and ThermoFisher are provided on their official websites (ThermoFisher: www.thermofisher.com, Genewiz: www.genewiz.com). BiLSTM-CRF(b) has the highest average CAI, showing that it has great potential to enhance protein expression.

As shown in Table 3, the Jaccard index^43^ was used to measure the similarities of the optimized sequence from BiLSTM-CRF(b) with the original sequence and the sequences from Genewiz, ThermoFisher, and BiLSTM-CRF(a). The average 20%-28% difference between BiLSTM-CRF(b) and ThermoFisher or Genewiz shows that our method is a new approach for discovering underlying features of data, and it is different from BiLSTM-CRF(a).

**Table 3:** Comparative analysis of Jaccard similarity.

DNA	Original	Genewiz	ThermoFisher	BiLSTM-CRF(a)
PTP4A3	0.68	0.74	0.80	0.85
PA	0.62	0.72	0.82	0.90
PAE	0.70	0.70	0.79	0.90
FALVAC-1	0.62	0.73	0.80	0.88
HPDF	0.70	0.73	0.80	0.90
MMPL3	0.65	0.69	0.76	0.89
Average	0.66	0.72	0.80	0.89

Jaccard similarity index between the optimized sequences of BiLSTM-CRF(b) and others.

### Experimental results for FALVAC-1 and PTP4A3

**Figure 3:**
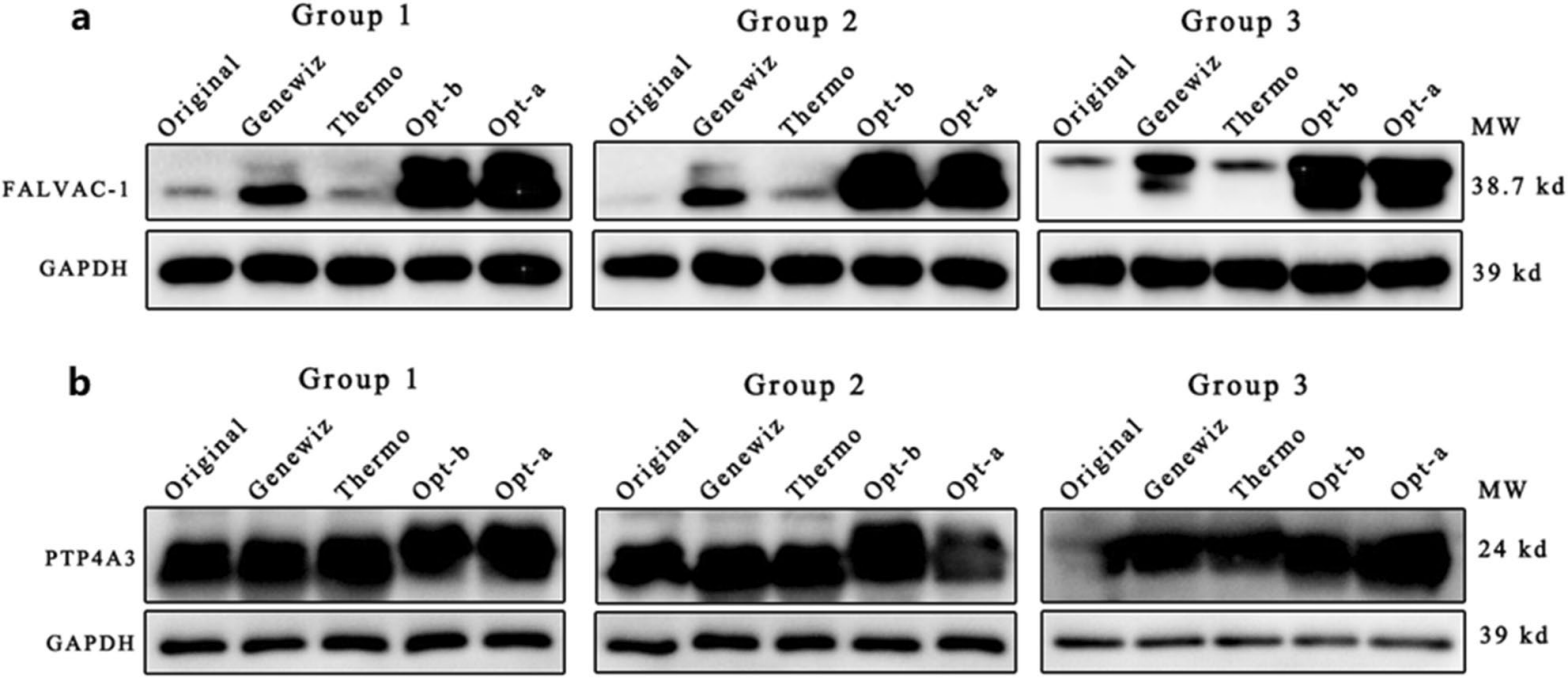
Comparison of protein expression levels for FALVAC-1 and PTP4A3. (**a**) shows the results of western blotting for FALVAC-1. (**b**) shows the results of western blotting for PTP4A3.

Because the CAI is simply a factor that affects protein expression, to further validate the rationality of our codon optimization method, the FALVAC-1 protein (FALVAC-1 was constructed as a multivalent plasmodium falciparum vaccine antigen and expressed in *E. coli*) and PTP4A3 protein were expressed in *E. coli,* and their expression levels were analyzed by western blot analysis. We compared three groups (Group 1, Group 2 and Group 3) of parallel experiments simultaneously for FALVAC-1 and PTP4A3, and compared the optimization effects of five sequences (namely, Original, Genewiz, Thermo, Opt-b and Opt-a, where Opt-b stands for BiLSTM-CRF(b) and Opt-a stands for BiLSTM-CRF(a)) in each group. The comparison of protein expression levels is shown in Fig. 3.

As shown in Fig. 3a and the corresponding Table 4, according to the optimization ratio, protein expression from the FALVAC-1 gene sequence optimized by Opt-b was significantly better than that obtained with the other methods. Furthermore, the protein expression obtained with Opt-b was better than that obtained with Opt-a, which indicates that the introduction of a codon box is necessary and useful. As shown in Fig. 3b and the corresponding Table 5, when the original sequence is well expressed, although Opt-b is still the best method, the optimization ratio is not very significant, and all methods have approximately equal optimization ratios. The reason for this result is that the codon distribution of PTP4A3 is similar to that of *E. coli* genes. Hence, the result indicates that the new model is robust and reflects the distribution of host genes. Moreover, the experimental results are also clearly consistent with the theoretical predictions based on the CAI in Table 2.

**Table 4:** Comparison of grayscale value ratios corresponding to Fig. 3a.

	Original	Genewiz	Thermo	Opt-b	Opt-a
Group 1	0.221	0.875	0.548	2.178	1.669
Group 2	0.090	0.742	0.352	2.115	1.747
Group 3	0.245	0.901	0.331	1.935	1.762
Average value	0.186	0.839	0.410	2.076	1.726
Optimization ratio	1	4.511	2.204	11.129	9.462

The comparison of grayscale value ratios between FALVAC-1 and GAPDH. The optimization ratio is the ratio of each method's average value to the original average value.

**Table 5:** Comparison of grayscale value ratios corresponding to Fig. 3b.

	Original	Genewiz	Thermo	Opt-b	Opt-a
Group 1	2.448	2.863	3.006	3.033	3.017
Group 2	3.398	3.506	3.564	4.568	3.266
Group 3	1.727	0.901	3.073	3.145	3.594
Average value	2.558	3.147	3.238	3.780	3.292
Optimization ratio	1	1.23	1.266	1.400	1.287

The comparison of grayscale value ratios between PTP4A3 and GAPDH. The optimization ratio is the ratio of each method's average value to the original average value.

In this paper, we chose two genes, namely, FALVAC-1 with a low expression level, which proved the effectiveness of our algorithm, and PTP4A3 with a high expression level, which proved the stability of our algorithm. FALVAC-1^45^ was constructed as a multivalent plasmodium falciparum vaccine antigen and expressed in *E. coli*, and PTP4A3^46^ was used as a negative control to prove that our algorithm will not cause low expression.

**Figure 4:**
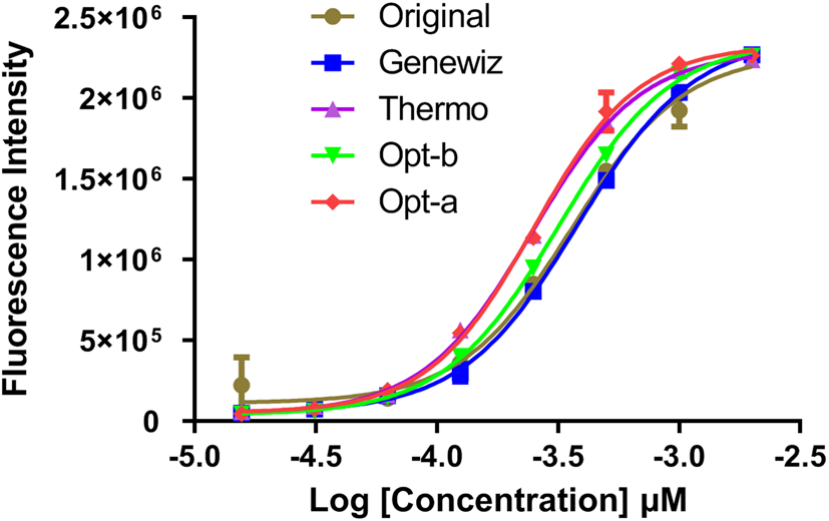
The assay of protein function for PTP4A3. In vitro phosphatase assays showed that the activities of proteins expressed by five sequences were almost equal (where p > 0.05). Different sequences are represented by different colors.

Furthermore, according to the method reported in the literature, the activities of each purified protein were detected by more experiments, as shown in Fig. 4. No significant difference in the protein's activity among the five sequences (Original, Genewiz, Thermo, Opt-b and Opt-a) was demonstrated. This result proved that our optimization had no effect on the protein's function.

## Discussion

In this paper, we introduce the concept of codon boxes, via which DNA sequences can be recoded into codon box sequences while ignoring the order of bases. Then, the problem of codon optimization can be converted to sequence annotation of corresponding amino acids with codon boxes. Because deep learning is a good method to obtain the distribution characteristics of DNA sequences, it is theoretically more advantageous for tackling codon optimization than existing index optimization methods. According to the results of biological experiments, compared with the codon optimization tools that are widely used in the industry, our method is likely competitive in terms of genetic engineering. Our optimization model was originally designed for *E. coli* in this paper, while its generality for other species, such as insect cells and yeasts needs further research in the future. Moreover, with the development of deep learning, the optimization method can be further improved to obtain better protein expression.

In fact, codon optimization can also be regarded as an inverse problem of codon sequences coding amino acid sequences. However, the inverse problem is not one-to-one mapping, and whether a learning mechanism for amino acid sequence to DNA sequence conversion exists is not clear. Because the deep neural network is a black box, the underlying biological reasons cannot be adequately explained at the present time. Therefore, it is reasonably expected that other experts will provide further biological insights into the learning mechanism in the future.

## Methods

### Codon optimization

To obtain the final optimized sequence, we implemented a bidirectional long/short-term memory neural network with a conditional random field layer (BiLSTM-CRF)^44^ that is able to annotate amino acid sequences with codons or codon boxes. First, the codon sequences can be decoded into the corresponding amino acid sequence. The word-embedding vectors of amino acid sequences are regarded as inputs of BiLSTM-CRF. The model parameters were iteratively optimized on the training set using *L2* regularization, and the model with the best performance on the validation set was chosen. BiLSTM-CRF provides each amino acid and its annotated codon or codon box token as the output. Because a codon box and an amino acid can be used to uniquely determine a codon, the optimized codon sequence can be obtained.

#### CAI

CAI is calculated as per formula (1):

1 $$ CAI = \left( {\prod\limits_{k = 1}^{L} {w_{k} ,} } \right) $$

where *L* is the number of codons, an *w*_*k*_ is calculated as per formula (2):

2 $$ w_{k} = \frac{{RSCU_{i} }}{{RSCU_{\max } }} $$

where *RSCU*_max_ is the highest codon usage frequency for synonymous codons in highly expressed reference gene and *RSCU*_*i*_ is the relative frequency of the unified codon of the first codon encoding the corresponding amino acids.

### Protein expression

The original gene and optimized codons of PTP4A3 and FALVAC-1 were subcloned into the pET28a(+) vector with a hexahistidine affinity tag fused to the N terminus and transformed into *E.coli*(BL21(DE3)). All plasmids were ordered from Genewiz (www.genewiz.com/). Upon bacterial growth to an optical density of 0.6–0.8 at 600 nm in lysogeny broth containing 50 µg/ml kanamycin at 37 °C in a shaker at 220 rpm, induction was carried out at 16 °C using 0.2 mM isopropyl-b-D-thiogalactoside (IPTG), and growth was continued at 16 °C for approximately 18 h. The cells were harvested by centrifugation and stored at − 80 °C or used for the subsequent steps.

Harvested cells were resuspended in lysis buffer (PBS). M protease inhibitor (phenylmethanesulfonyl fluoride, PMSF) was added to the cell sample before lysis, and then, the cell lysate was obtained by ultrasonication. The cell lysate was centrifuged at 15,000 rpm for 45 min, after which the supernatant was collected. The protein concentration was determined by the Beyotime BCA Protein Assay Kit.

Then, a western blot analysis was carried out on the extracted samples, with anti-His as the primary antibody. Primary antibody incubation was followed by probing with the corresponding secondary antibody, and the blot was developed using Image Lab Touch Software.

## Supplementary information

Supplementary Information.
