# Supplementary material for: Codon optimization with deep learning to enhance protein expression
Source: Sci Rep. 2020 Oct 19;10:17617. doi: 10.1038/s41598-020-74091-z (PMC7572362; doi:10.1038/s41598-020-74091-z)
Supplement: Supplementary file 1 — Supplementary Information. [file 41598_2020_74091_MOESM1_ESM.docx]

**Codon Optimization with Deep Learning to Enhance Protein Expression**

**Hongguang Fu^1^, Yanbing Liang^1^, Xiuqin Zhong^1,*^, ZhiLing Pan^2^, Lei Huang^1^, HaiLin Zhang^2^, Yang Xu^1^, Wei Zhou^1^ and Zhong Liu^3^**

1. University of Electronic Science and Technology of China, Chengdu, 611731, China;

2. State Key Laboratory of Biotherapy, West China Hospital, Sichuan University, Chengdu, 610041, China;

3.Chengdu Institute of Computer Applications, Chinese Academy of Sciences, Chengdu, 610041, China

*[zhongxiuqin2009@gmail.com](mailto:zhongxiuqin2009@gmail.com)

**Original images for figure 3**

Correspond_To_Fig3a_FALVAC-1_Group 1 and 2


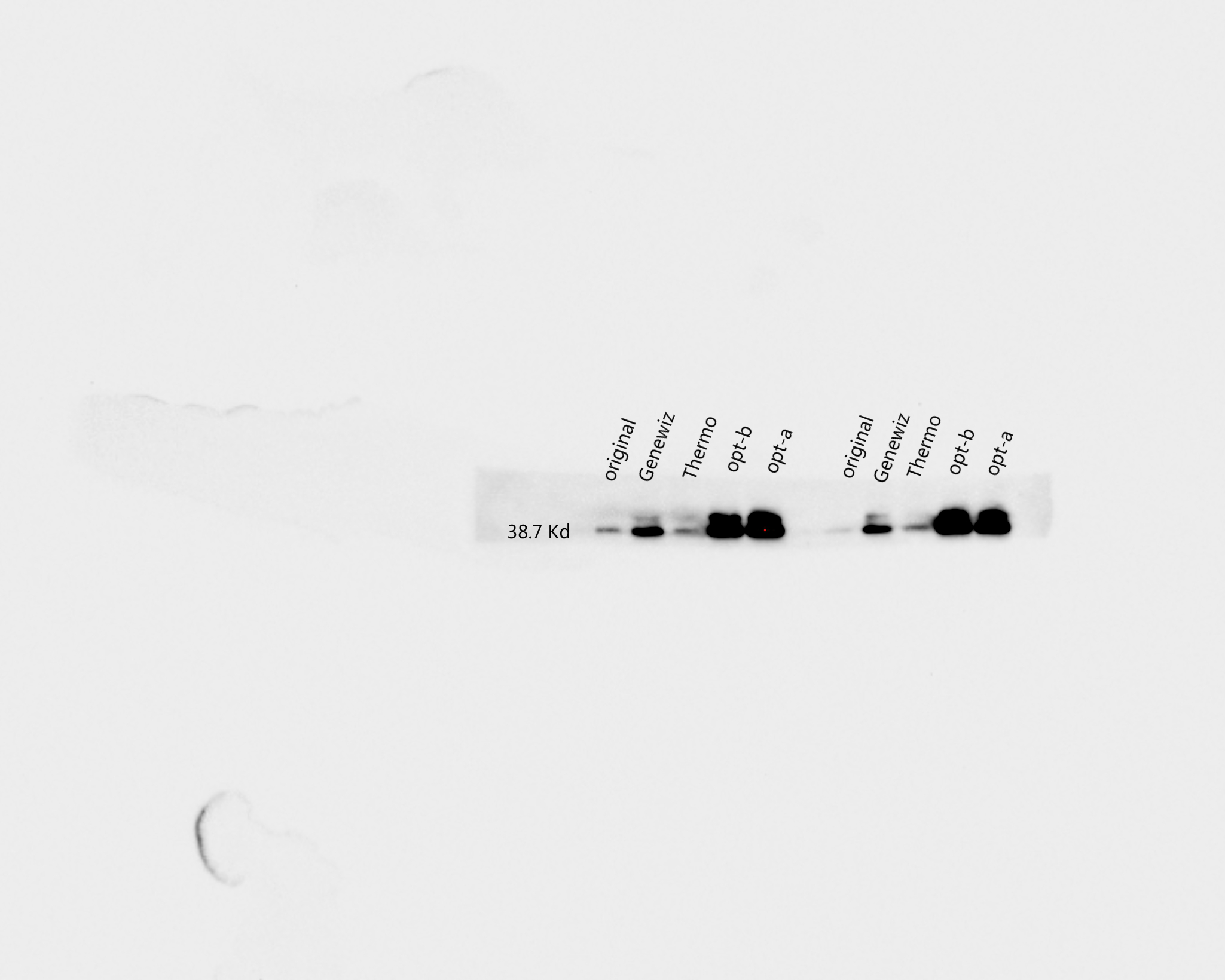


Correspond_To_Fig3a_FALVAC-1_Group 3


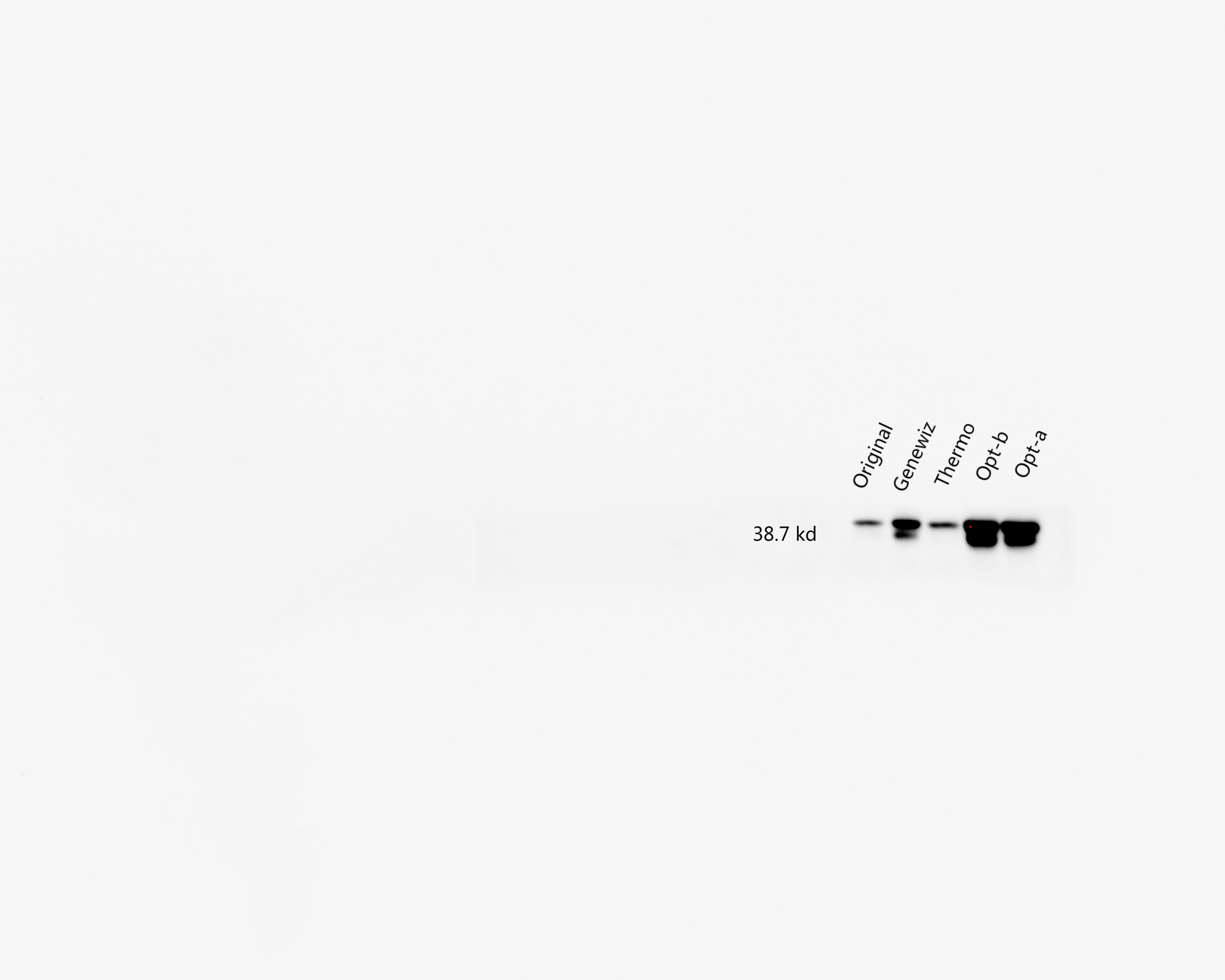


Correspond_To_Fig3a_GAPDH_Group 1 and 2


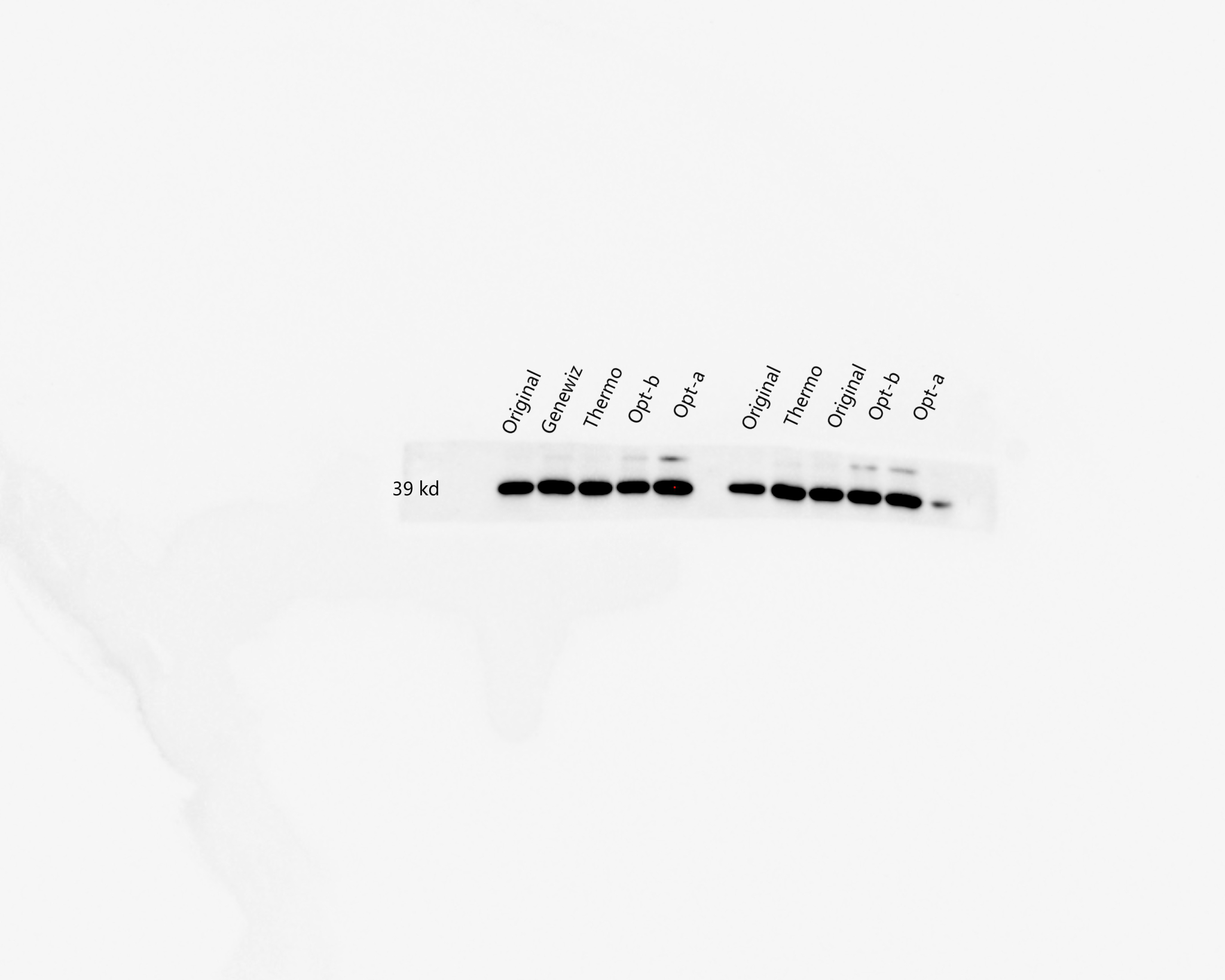


Correspond_To_Fig3a_GAPDH_Group 3


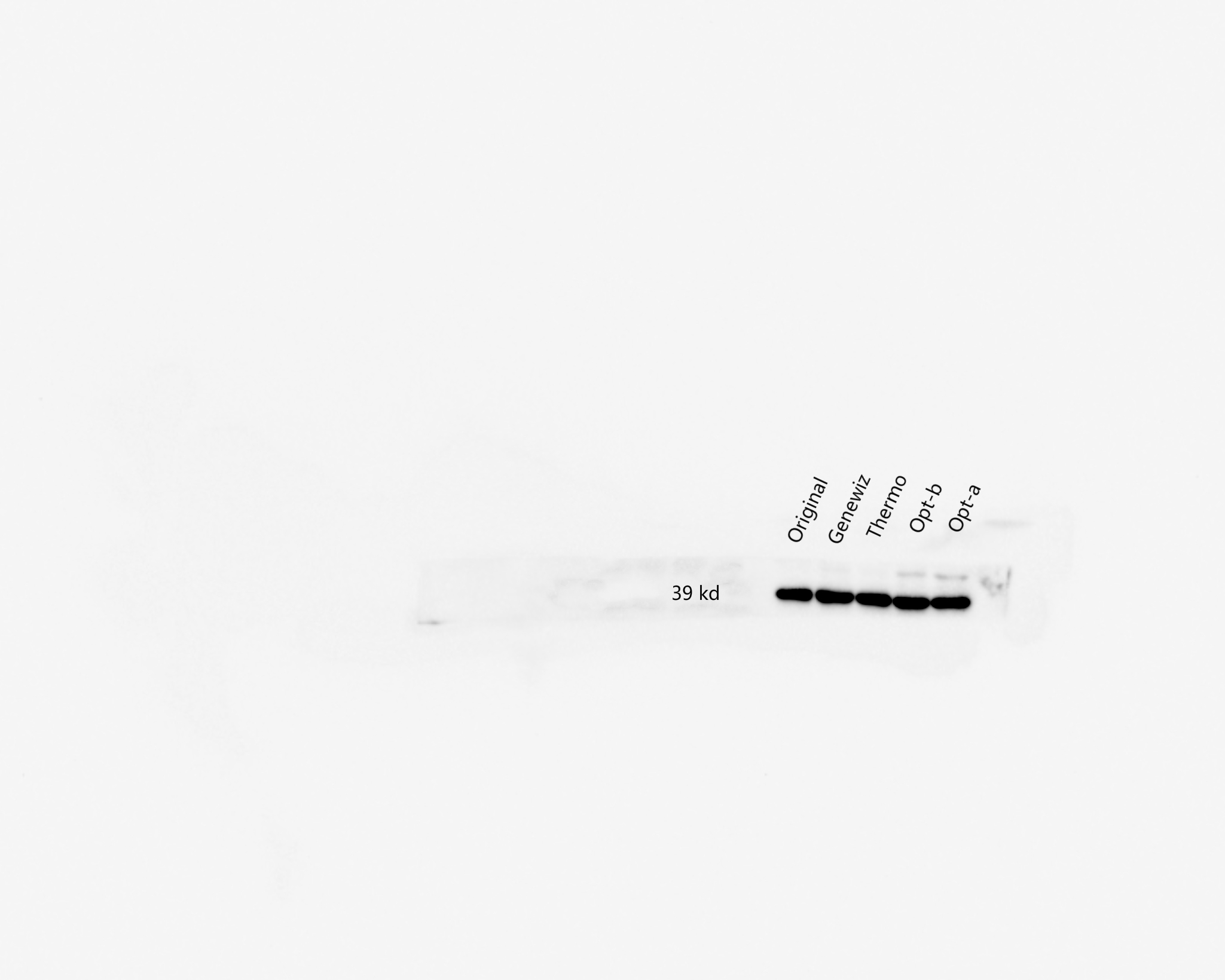


Correspond_To_Fig3b_GAPDH_Group 1 and 2


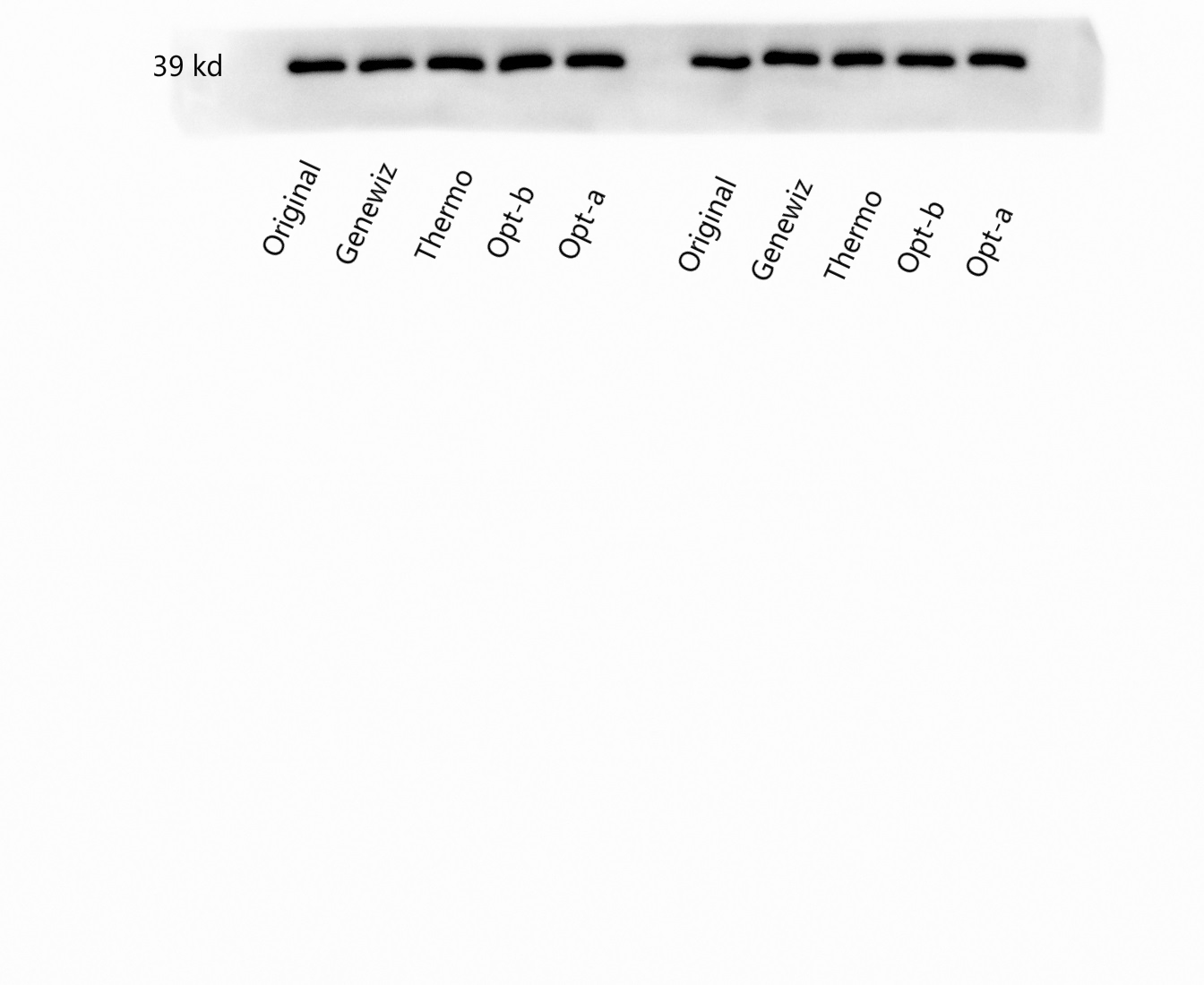


Correspond_To_Fig3b_GAPDH_Group 3


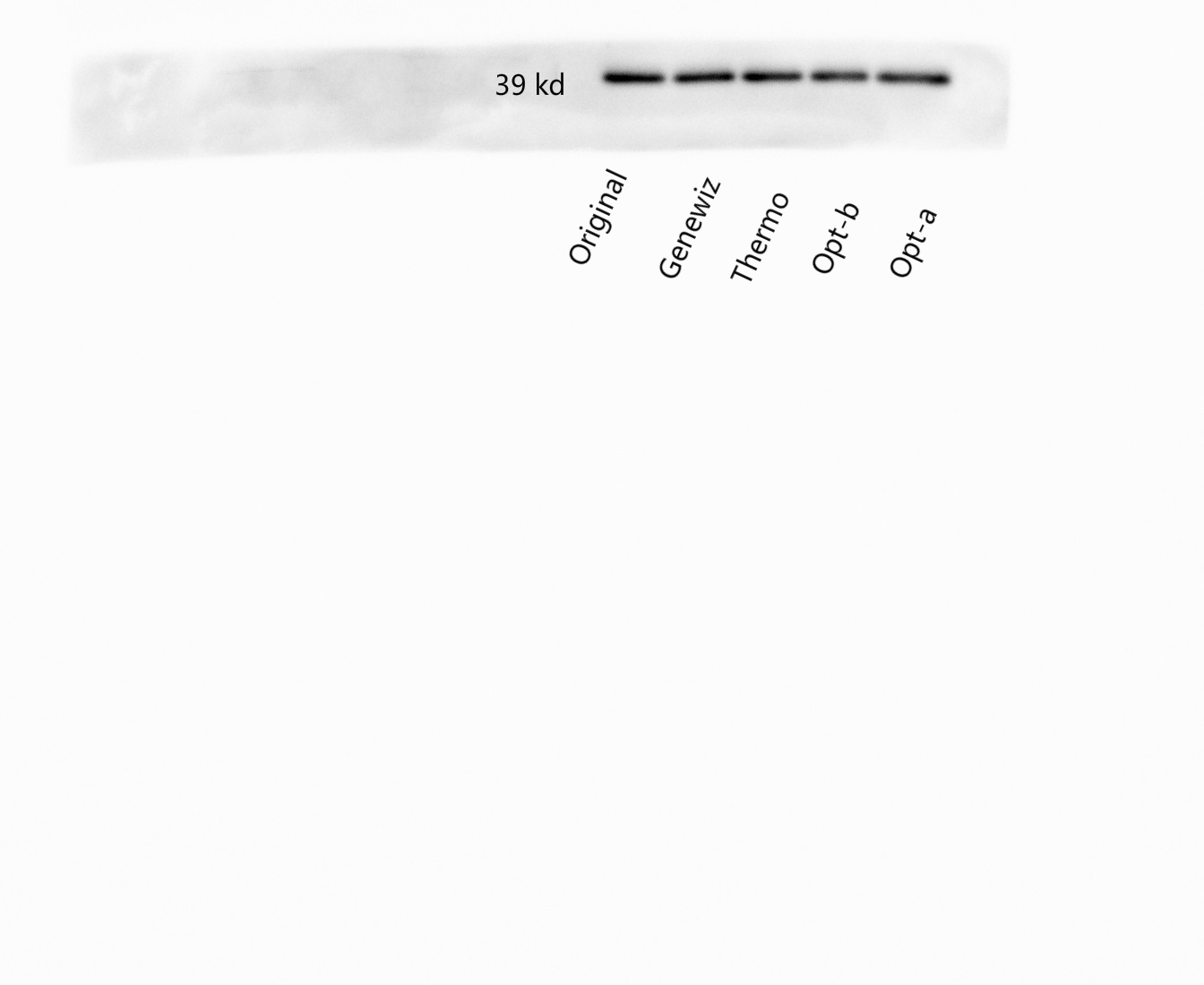


Correspond_To_Fig3b_PTP4A3_Group 1 and 2


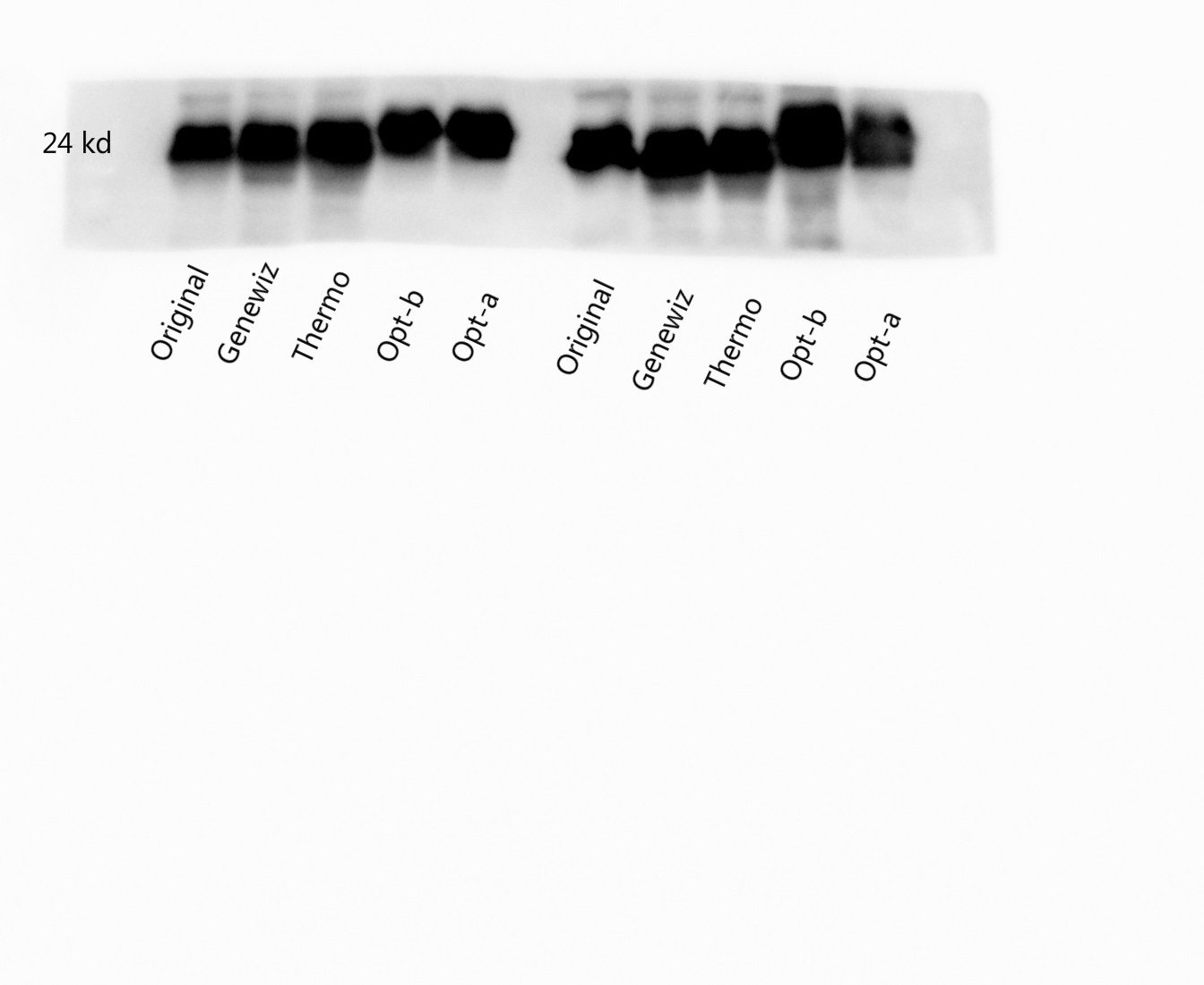


Correspond_To_Fig3b_PTP4A3_Group 3


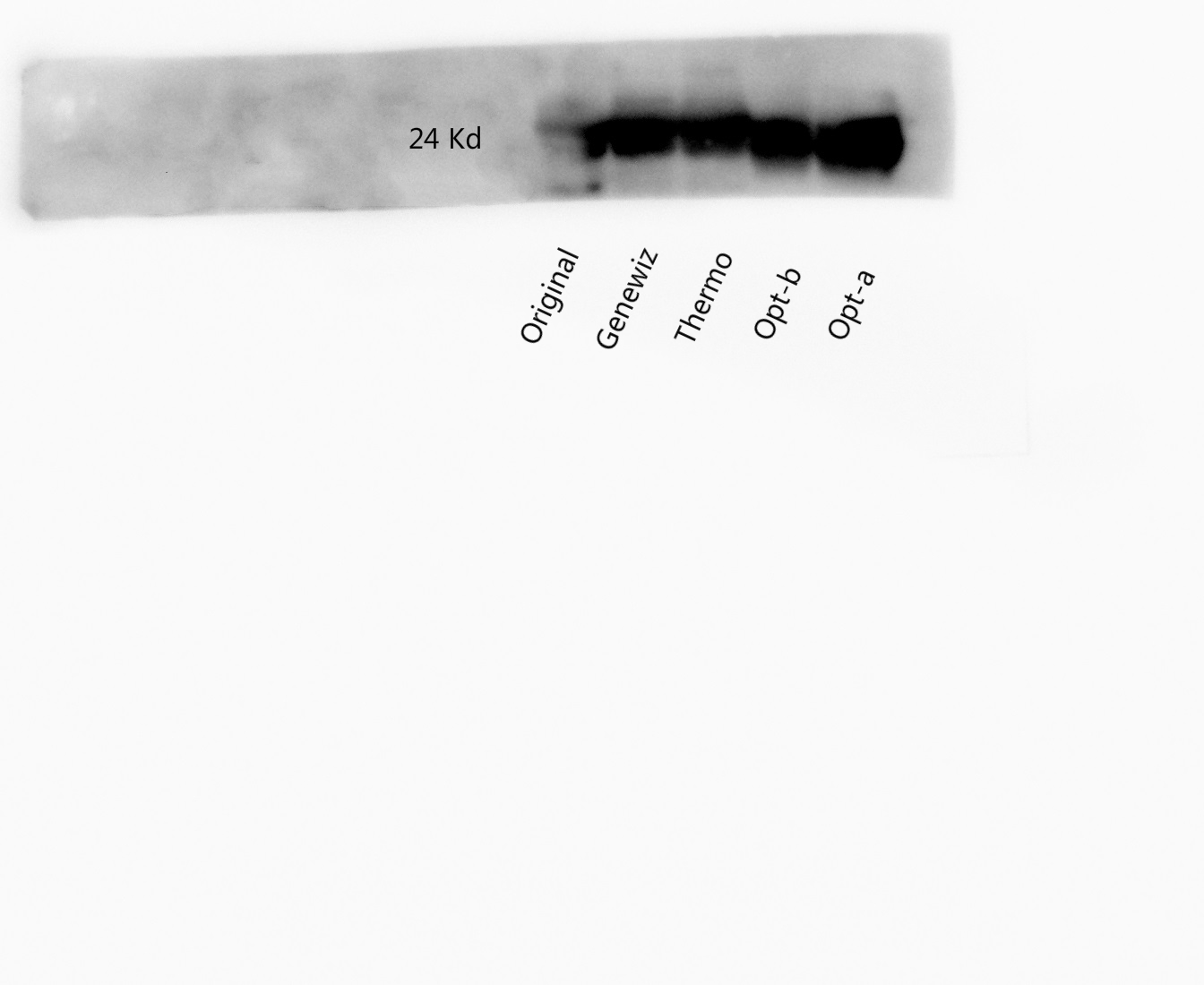


The antibody used in the experiment is Anti-His Tag.

Antibody source: The antibody was ordered from Absin, catalog number NA20.

Specificity of the antibody: This antibody can recognize the His-tag (HHHHHH) fused to either the amino or carboxy terminal of targeted protein.

**Experimental data:**
**FALVAC-1**

**Original**

ATGGCTAAACATAAAAAATTAAAGCAACCAGGGGATGGTAATCCTTGGTCCCCATGTAGTGTAACTTGTGGAAAACCTAAAGACGAATTAGATTATGAAAATGATATTGAAAAAAAAATTTGTAAAATGGAAAAATGTTCCAGTGTGTTTAATGTCGTAAATAGTAATTCTGGATGTTTCAGACATTTAGATGAAAGAGAAGAATGTAAATGTTTATTAGAAGATTCAGGTAGCAACGGAAAGAAAATCACATGTGAATGTACTAAACCTGATTCTAAGCCTATTGTGCAATATGACAATTTCAATGCAAACCCAAACGCAAACCCCAATGCAAATCCTGATGGAAATTGTGAAGATATACCACATGTAAATGAATTTTCAGCAATTGATCTTGGAAATGCAGAAAAATATGATAAAATGGATGAACCACAACATTATGGGAAATCACTCACTCCATTAGAAGAATTATATAAACCAAATGATAAAAGTTTGTATCAGTATATAAAAGCAAATTCTAAATTTATAGGTATAACTGAACTAAGCAACACATTCATAAACAATGCTGGACAACATGGACATATGCATGGTAACGAGAGGGAAGATGAGAGAACGCTTACTAAGGAATATGAAGATATTGTTTTGAAAGAGTTTACATATATGATAAACTTTGGAAGAGGACAGAATTATTGGGAACATCCATATCAAAAAAGTGATCAACCTAAACAATATGAACAACATTTAACAGATTATGAAAAAATTAAAGAAGGTAAGCCCTTGGATAAATTTGGAAATATCTATGATTATCACTATGAGCATTCTAGTCCATCTAGTACAAAGTCATCAAGTCCATCAAATGTAAAATCAGCTAGTCTAGCTACAAGATTAATGAAAAAATTTAAAGCTGAAATCAGAGATTTCTTCGGTATAAGTTATTATGAAAAGGTTTTAGCGAAATATAAGGATGATTTAGAA

**Genewiz**

ATGGCGAAGCACAAAAAACTGAAGCAGCCGGGCGACGGTAACCCATGGAGTCCGTGTAGCGTGACGTGCGGCAAACCGAAGGATGAACTGGATTACGAAAATGATATCGAAAAAAAAATCTGCAAAATGGAAAAATGTAGCAGCGTGTTCAACGTGGTGAACAGCAATAGCGGCTGCTTTCGCCATCTGGATGAACGCGAGGAATGCAAATGTCTGCTGGAAGACAGTGGCAGCAACGGCAAGAAAATCACGTGCGAGTGCACGAAACCGGATAGCAAGCCGATCGTTCAGTACGATAATTTCAACGCCAACCCGAACGCCAACCCAAACGCGAATCCGGACGGTAACTGCGAGGATATCCCACATGTGAACGAGTTCAGCGCCATTGATCTGGGCAACGCGGAGAAGTACGACAAAATGGACGAGCCGCAGCACTACGGTAAAAGTCTCACCCCGCTCGAGGAGCTCTACAAGCCGAACGACAAGAGTCTGTACCAGTACATTAAGGCGAACAGCAAATTCATCGGCATTACGGAGCTGAGCAACACCTTCATCAATAACGCCGGCCAACACGGTCATATGCACGGTAACGAACGCGAGGATGAACGCACGCTCACCAAAGAGTACGAGGACATCGTGCTGAAGGAGTTTACGTACATGATCAATTTCGGCCGCGGCCAGAACTATTGGGAGCACCCGTACCAGAAGAGCGACCAGCCAAAGCAGTACGAGCAGCATCTGACGGACTACGAAAAGATCAAGGAAGGCAAGCCGCTGGACAAGTTTGGCAACATTTACGACTACCACTACGAACACAGCAGCCCGAGCAGCACCAAAAGCAGTAGCCCGAGCAACGTTAAGAGCGCGAGTCTGGCGACCCGTCTGATGAAGAAGTTCAAGGCCGAAATCCGCGACTTCTTTGGCATCAGCTACTACGAGAAGGTGCTGGCCAAGTACAAGGATGACCTCGAA

**ThermoFisher**

ATGGCCAAACACAAAAAACTGAAACAGCCTGGTGATGGTAATCCGTGGTCACCGTGTAGCGTTACCTGTGGTAAACCGAAAGATGAACTGGATTATGAGAACGACATCGAGAAAAAGATCTGCAAGATGGAAAAATGCAGCAGCGTTTTTAATGTGGTGAATAGCAATAGCGGTTGCTTTCGTCATCTGGATGAACGTGAAGAATGTAAATGTCTGCTGGAAGATAGCGGTAGCAATGGCAAAAAAATCACCTGTGAATGTACCAAACCGGATAGCAAACCGATTGTTCAGTATGATAACTTTAACGCCAATCCGAATGCAAATCCTAATGCCAATCCGGATGGTAACTGTGAAGATATTCCGCATGTTAATGAATTCAGCGCAATTGATCTGGGCAACGCCGAAAAATATGATAAAATGGATGAACCGCAGCACTACGGTAAAAGCCTGACACCGCTGGAAGAACTGTATAAACCGAATGATAAATCCCTGTATCAGTATATCAAAGCCAACAGCAAATTTATCGGCATTACCGAACTGAGCAACACCTTTATTAACAATGCAGGTCAGCATGGTCATATGCATGGTAATGAACGCGAAGATGAACGTACCCTGACCAAAGAATATGAAGATATCGTGCTGAAAGAATTTACCTACATGATCAATTTTGGCCGTGGCCAGAATTATTGGGAACATCCGTATCAGAAAAGCGATCAGCCGAAACAGTATGAACAGCATCTGACCGATTACGAGAAAATCAAAGAAGGTAAACCGCTGGATAAATTCGGCAACATCTATGATTATCACTATGAACATAGCAGCCCGAGCAGCACCAAAAGCAGCAGCCCGTCAAATGTTAAAAGCGCAAGCCTGGCAACCCGTCTGATGAAAAAATTCAAAGCAGAAATCCGCGACTTTTTCGGCATCAGCTATTATGAAAAAGTGCTGGCGAAATACAAAGACGACCTGGAA

**BiLSTM-CRF(a)**

ATGGCGAAACATAAAAAACTGAAACAGCCGGGCGATGGTAACCCGTGGTCGCCGTGCAGCGTCACCTGCGGTAAACCGAAAGATGAACTGGATTATGAAAACGATATTGAAAAAAAAATTTGTAAAATGGAAAAATGTTCCAGCGTCTTTAACGTGGTCAACAGCAACAGCGGCTGCTTCCGCCATCTGGATGAACGTGAAGAGTGTAAATGCCTGCTGGAAGATTCCGGCAGCAACGGTAAAAAAATCACCTGTGAATGTACCAAACCGGATAGCAAACCGATTGTGCAGTATGATAACTTTAACGCCAACCCGAACGCCAACCCGAACGCCAACCCGGATGGTAACTGTGAAGATATTCCGCATGTCAACGAATTCAGCGCCATTGATCTGGGTAACGCGGAAAAATATGATAAAATGGATGAACCGCAACATTACGGTAAATCGCTGACGCCGCTGGAAGAGCTGTATAAACCGAACGATAAATCGCTGTATCAGTATATTAAAGCCAACAGCAAATTTATTGGCATTACCGAACTGAGCAACACCTTTATTAACAACGCCGGTCAGCATGGTCATATGCACGGTAACGAACGTGAAGATGAACGTACGCTGACCAAAGAATATGAAGATATTGTGCTGAAAGAGTTTACCTATATGATTAACTTTGGTCGTGGTCAGAACTACTGGGAACATCCGTATCAGAAAAGCGATCAGCCGAAACAATATGAACAACATCTGACCGATTATGAAAAAATCAAAGAAGGTAAACCGCTGGATAAATTTGGTAACATTTATGATTATCATTATGAACATTCCAGCCCGTCCAGCACCAAATCCAGCAGCCCGAGCAACGTGAAAAGCGCCAGCCTGGCGACGCGTCTGATGAAAAAATTTAAAGCGGAAATTCGCGATTTCTTTGGTATCAGCTATTATGAAAAAGTGCTGGCGAAATATAAAGATGATCTGGAA

**BiLSTM-CRF(b)**

ATGGCGAAACATAAAAAACTGAAACAGCCGGGTGATGGCAACCCGTGGAGCCCGTGCAGCGTGACGTGCGGTAAACCGAAAGATGAACTGGATTATGAAAACGATATTGAAAAAAAAATTTGCAAAATGGAAAAATGCAGCAGCGTGTTTAACGTGGTTAACAGCAACAGCGGCTGCTTTCGCCATCTGGATGAACGTGAAGAATGCAAATGCCTGCTGGAAGATAGCGGCAGCAACGGTAAAAAAATTACCTGCGAATGCACCAAACCGGATAGCAAACCGATTGTTCAGTATGATAACTTTAACGCTAACCCGAACGCTAACCCGAACGCTAACCCGGATGGCAACTGCGAAGATATTCCGCATGTTAACGAGTTTTCCGCGATTGATCTGGGCAACGCGGAAAAATACGATAAAATGGATGAACCGCAGCATTACGGTAAAAGCCTGACGCCGCTGGAAGAGCTGTATAAACCGAACGATAAAAGCCTGTATCAGTATATCAAAGCGAACAGCAAATTTATTGGTATTACCGAACTGAGCAACACCTTTATTAACAACGCCGGTCAGCACGGTCATATGCACGGTAACGAACGTGAAGATGAACGCACGCTGACCAAAGAATATGAAGATATTGTGCTGAAAGAGTTTACCTATATGATTAACTTTGGTCGCGGTCAGAACTACTGGGAACATCCGTATCAGAAAAGCGATCAGCCGAAACAGTATGAACAACATCTGACCGATTATGAAAAAATCAAAGAAGGTAAACCGCTGGATAAATTTGGTAACATTTACGATTATCATTATGAACATAGCAGCCCGAGCAGCACCAAAAGCAGCAGCCCGAGCAACGTGAAAAGCGCGAGCCTGGCGACGCGTCTGATGAAAAAATTTAAAGCGGAAATTCGCGATTTTTTTGGTATTTCCTATTATGAAAAAGTGCTGGCGAAATACAAAGATGATCTGGAA

**PTP4A3**

**Original**

AGCAGCCATCACCATCATCACCATAGCAGCGGTGAGAATCTGTACTTCCAAGGCATGGCTCGGATGAACCGCCCGGCCCCGGTGGAGGTGAGCTACAAACACATGCGCTTCCTCATCACCCACAACCCCACCAACGCCACGCTCAGCACCTTCATTGAGGACCTGAAGAAGTACGGGGCTACCACTGTGGTGCGTGTGTGTGAAGTGACCTATGACAAAACGCCGCTGGAGAAGGATGGCATCACCGTTGTGGACTGGCCGTTTGACGATGGGGCGCCCCCGCCCGGCAAGGTAGTGGAAGACTGGCTGAGCCTGGTGAAGGCCAAGTTCTGTGAGGCCCCCGGCAGCTGCGTGGCTGTGCACTGCGTGGCGGGCCTGGGCCGGGCTCCAGTCCTTGTGGCGCTGGCCCTTATTGAGAGCGGGATGAAGTACGAGGACGCCATCCAGTTCATCCGCCAGAAGCGCCGCGGAGCCATCAACAGCAAGCAGCTCACCTACCTGGAGAAATACCGGCCCAAACAGAGGCTGCGGTTCAAAGACCCACACACGCACAAGACCCGG

**Genewiz**

AGCAGCCATCACCATCATCACCATAGCAGCGGTGAGAATCTGTACTTCCAAGGCATGGCCCGTATGAATCGCCCGGCGCCGGTTGAGGTGAGCTACAAGCACATGCGCTTTCTGATCACGCACAACCCGACCAACGCCACGCTGAGCACCTTCATCGAGGATCTGAAGAAGTACGGTGCCACCACGGTTGTGCGCGTGTGCGAAGTGACCTATGACAAAACCCCGCTCGAGAAGGATGGCATCACCGTTGTGGATTGGCCATTCGATGACGGTGCGCCACCGCCGGGCAAAGTTGTTGAAGACTGGCTGAGTCTGGTGAAGGCGAAATTTTGCGAAGCGCCGGGTAGCTGCGTTGCCGTTCATTGCGTTGCCGGTCTGGGTCGTGCGCCAGTTCTGGTTGCGCTGGCGCTGATCGAGAGTGGCATGAAGTACGAGGACGCGATTCAGTTCATCCGCCAGAAACGCCGCGGCGCGATCAACAGCAAACAGCTCACCTATCTGGAGAAGTACCGCCCAAAACAGCGTCTGCGCTTTAAAGACCCGCACACGCACAAAACGCGC

**ThermoFisher**

AGCAGCCATCACCATCATCATCATAGCAGCGGTGAAAACCTGTATTTTCAAGGTATGGCACGTATGAATCGTCCGGCACCGGTTGAAGTTAGCTATAAACATATGCGTTTTCTGATCACCCATAATCCGACCAATGCAACCCTGAGCACCTTTATTGAAGATCTGAAAAAATACGGTGCCACCACCGTTGTTCGTGTTTGTGAAGTTACCTATGATAAAACACCGCTGGAAAAAGATGGTATTACCGTTGTTGATTGGCCGTTTGATGATGGCGCACCGCCTCCGGGTAAAGTTGTTGAAGATTGGCTGAGCCTGGTTAAAGCCAAATTTTGTGAAGCACCGGGTAGCTGTGTTGCAGTTCATTGTGTTGCCGGTCTGGGTCGTGCACCGGTTCTGGTTGCACTGGCACTGATTGAAAGCGGTATGAAATATGAAGATGCCATCCAGTTTATTCGTCAGAAACGTCGTGGTGCCATTAATAGCAAACAGCTGACCTATCTGGAAAAGTATCGTCCGAAACAGCGTCTGCGTTTTAAAGATCCGCATACACATAAAACCCGC

**BiLSTM-CRF(a)**

TCCAGCCATCATCATCATCATCACAGCAGCGGTGAAAACCTGTATTTCCAGGGCATGGCGCGTATGAACCGTCCGGCGCCGGTGGAAGTCAGCTATAAACATATGCGTTTTCTGATTACCCATAACCCGACCAACGCCACGCTGTCCACCTTTATTGAAGATCTGAAAAAATATGGCGCGACCACCGTGGTGCGTGTCTGCGAAGTCACCTATGATAAAACGCCGCTGGAAAAAGATGGCATTACCGTGGTGGATTGGCCGTTTGATGATGGCGCGCCGCCGCCGGGTAAAGTGGTGGAAGATTGGCTGTCGCTGGTGAAAGCGAAATTCTGTGAAGCGCCGGGTTCCTGCGTGGCGGTGCATTGCGTGGCGGGTCTGGGTCGTGCGCCGGTGCTGGTGGCGCTGGCGCTGATTGAAAGCGGCATGAAATATGAAGATGCGATTCAGTTTATTCGTCAGAAACGCCGCGGCGCGATTAACAGCAAACAACTGACCTATCTGGAAAAATATCGCCCGAAACAGCGTCTGCGTTTTAAAGATCCGCATACCCATAAAACCCGT

**BiLSTM-CRF(b)**

AGTAGCCATCATCATCATCATCATTCCAGCGGCGAAAACCTGTATTTTCAGGGCATGGCGCGTATGAACCGCCCGGCACCGGTTGAAGTGAGCTATAAACATATGCGTTTTCTGATTACCCATAACCCGACCAACGCCACGCTGAGCACCTTTATTGAAGATCTGAAAAAATACGGCGCGACCACCGTGGTTCGCGTGTGCGAAGTGACCTATGATAAAACGCCGCTGGAAAAAGATGGCATTACCGTGGTGGATTGGCCGTTTGATGATGGCGCGCCGCCGCCGGGTAAAGTGGTGGAAGATTGGCTGTCGCTGGTGAAAGCGAAATTTTGCGAAGCGCCGGGTAGCTGCGTGGCGGTGCATTGCGTGGCGGGTCTGGGCCGTGCGCCGGTGCTGGTGGCGCTGGCGCTGATTGAAAGCGGCATGAAATATGAAGATGCGATTCAGTTTATTCGCCAGAAACGCCGTGGCGCGATTAACAGCAAACAGCTGACCTATCTGGAAAAATATCGCCCGAAACAGCGTCTGCGTTTTAAAGATCCGCATACCCATAAAACCCGC

**PA**

**Original**

ATGGAAGACTTTGTGCGACAATGCTTCAATCCAATGATCGTCGAGCTTGCGGAAAAGGCAATGAAAGAATATGGGGAAGATCCGAAAATCGAAACTAACAAGTTTGCTGCAATATGCACACATTTGGAAGTTTGTTTCATGTATTCGGATTTCGGCTCTGGTGACCCGAATGCACTATTGAAGCACCGATTTGAGATAATTGAAGGAAGAGACCGAATCATGGCCTGGACAGTGGTGAACAGTATATGTAACACAACAGGGGTAGAGAAGCCTAAATTTCTTCCTGATTTGTATGATTACAAAGAGAACCGGTTCATTGAAATTGGAGTAACACGGAGGGAAGTCCACATATATTACCTAGAGAAAGCCAACAAAATAAAATCTGAGAAGACACACATTCACATCTTTTCATTCACTGGAGAGGAGATGGCCACCAAAGCGGACTACACCCTTGACGAAGAGAGCAGGGCAAGAATCAAAACTAGGCTTTTCACTATAAGACAAGAAATGGCCAGTAGGAGTCTATGGGATTCCTTTCGTCAGTCCGAAAGAGGCGAATAA

**Genewiz**

ATGGAAGACTTCGTGCGCCAATGCTTCAACCCGATGATCGTGGAGCTGGCCGAGAAAGCGATGAAAGAATACGGCGAGGACCCGAAGATCGAGACCAACAAATTCGCGGCCATCTGCACCCATCTCGAAGTGTGCTTCATGTACAGCGACTTCGGTAGCGGCGATCCGAATGCGCTGCTCAAGCACCGTTTTGAGATCATCGAGGGTCGCGATCGCATCATGGCGTGGACCGTGGTGAACAGCATCTGCAATACGACGGGCGTGGAAAAGCCGAAATTTCTGCCGGATCTGTACGACTACAAGGAGAACCGCTTCATCGAAATCGGCGTGACCCGCCGCGAGGTGCACATCTACTATCTGGAAAAGGCCAATAAAATCAAAAGCGAGAAAACGCACATCCATATCTTCAGCTTCACCGGCGAAGAAATGGCCACCAAAGCGGATTACACGCTGGATGAGGAAAGCCGCGCGCGTATCAAAACGCGTCTGTTCACCATCCGCCAAGAAATGGCGAGTCGTAGTCTGTGGGACAGTTTCCGCCAGAGCGAACGCGGCGAATAA

**ThermoFisher**

ATGGAAGATTTTGTGCGCCAGTGTTTTAATCCGATGATTGTTGAACTGGCAGAGAAGGCCATGAAAGAATATGGTGAAGATCCGAAAATCGAAACCAACAAATTTGCAGCCATTTGCACCCATCTGGAAGTGTGTTTTATGTATAGCGATTTTGGTAGCGGTGATCCGAATGCACTGCTGAAACATCGTTTTGAAATTATCGAAGGTCGCGATCGTATTATGGCATGGACCGTTGTTAATAGCATTTGTAATACCACCGGTGTGGAAAAACCGAAATTTCTGCCGGATCTGTATGACTATAAAGAGAACCGCTTTATTGAAATTGGTGTGACCCGTCGTGAAGTGCATATTTACTATCTGGAAAAAGCCAACAAGATCAAAAGCGAGAAAACCCACATTCACATCTTTAGCTTTACCGGTGAAGAAATGGCAACCAAAGCAGATTATACCCTGGATGAAGAAAGCCGTGCACGTATTAAAACCCGTCTGTTTACCATTCGTCAAGAGATGGCAAGCCGTAGCCTGTGGGATAGCTTTCGTCAGAGCGAACGTGGTGAATAA

**BiLSTM-CRF（a）**

ATGGAAGATTTTGTCCGTCAGTGCTTTAACCCGATGATTGTCGAACTGGCGGAAAAAGCGATGAAAGAATATGGCGAAGATCCGAAAATTGAAACCAACAAATTTGCGGCGATTTGTACCCATCTGGAAGTCTGCTTTATGTATAGCGATTTTGGTTCCGGCGATCCGAACGCGCTGCTGAAACATCGTTTTGAAATTATTGAAGGTCGCGATCGCATTATGGCGTGGACCGTGGTCAACAGCATTTGTAACACCACCGGCGTGGAAAAACCGAAATTCCTGCCGGATCTGTATGATTATAAAGAAAACCGTTTTATTGAAATTGGCGTCACCCGCCGTGAAGTGCATATTTATTATCTGGAAAAAGCCAACAAAATCAAAAGCGAAAAAACCCATATTCATATTTTCAGTTTTACCGGTGAAGAAATGGCGACCAAAGCGGATTATACCCTGGATGAAGAAAGCCGTGCGCGTATTAAAACCCGTCTGTTTACCATTCGTCAGGAAATGGCGAGCCGTTCGCTGTGGGATAGCTTCCGTCAGAGCGAACGCGGTGAATAA

**BiLSTM-CRF（b）**

ATGGAAGATTTTGTTCGTCAGTGCTTTAACCCGATGATTGTCGAACTGGCGGAAAAAGCGATGAAAGAATATGGTGAAGATCCGAAAATTGAAACCAACAAATTTGCGGCGATTTGCACCCATCTGGAAGTGTGCTTTATGTATTCCGATTTTGGTAGCGGCGATCCGAACGCGCTGCTGAAACATCGCTTTGAAATTATTGAAGGCCGTGATCGTATTATGGCGTGGACCGTGGTTAACAGCATTTGCAACACCACCGGCGTGGAAAAACCGAAATTTCTGCCGGATCTGTATGATTACAAAGAAAACCGTTTTATTGAAATTGGCGTTACCCGCCGTGAAGTGCATATTTATTATCTGGAAAAAGCGAACAAAATCAAAAGCGAAAAAACCCATATCCATATTTTTTCTTTTACCGGTGAAGAAATGGCGACCAAAGCGGATTATACCCTGGATGAAGAAAGCCGTGCGCGTATTAAAACCCGCCTGTTTACCATTCGCCAGGAAATGGCGAGCCGCAGCCTGTGGGATAGCTTTCGCCAGAGCGAACGCGGTGAATAA

**PAE：**

**Original**

GCCGAGGAAGCCTTCGACCTGTGGAACGAATGCGCCAAGGCCTGCGTGCTCGACCTCAAGGACGGCGTGCGTTCCAGCCGCATGAGCGTCGACCCGGCCATCGCCGACACCAACGGCCAGGGCGTGCTGCACCACTCCATGGTCCTGGAGGGCGGCAACGACGCGCTCAAGCTGGCCTTCGACAACGCCCTGAGCATCACCAGCGACGGCCTGACCATCCGCCTCGAAGGCGGCGTCGAGCCGAACAAGAAGGTGCGCTACAGCTACACGCGCCAGGCGCGCGGCAGTTGGTCGCTGAACTGGCTGGTACCGATCGGCCACGAGAAGCCCTCGAACATTAAGGTGTTCATCCACGAACTGAACGCCGGTAACCAGCTCAGCCACATGTCGCCGATCTACACCATCGAGATGGGCGACGAGTTGCTGGCGAAGCTGGCGCGCGATGCCACCTTCTTCGTCAGGGCGCACGAGAGCAACGAGATGCAGCCGACGCTCGCCATCAGCCATGCCGGGGTCAGCGTGGTCATGGCTCAGGCCCAGCCGCGCCGGGAAAAGCGCTGGAGCGAATGGGCCAGCGGCAAGGTGTTGTGCCTGCTCGACCCGCTGGACGGGGTCTACAACTACCTCGCCCAGCAGCGCTGCAACCTCGACGATACCTGGGAAGGCAAGATCTACCGGGTGCTCGCCGGCAACCCGGCGAAGCATGACCTGGACATCAAGCCCACGGTCATCAGTCATCGCCTGCATTTCCCCGAGGGCGGCAGCCTGGCAGCGCTGACCGCGCACCAGGCCTGCCACCTGCCGCTGGAGACCTTCACTCGTCATCGCCAGCCGCGCGGCTGGGAACAACTGGAGCAGTGCGGCTATCCGGTGCAGCGGCTGGTCGCCCTCTACCTGGCAGCGCGACTGTCGTGGAACCAGGTCGACCAGGTGATCCGCAACGCCCTGGCCAGCCCCGGTAGCGGCGGCGACCTGGGCGAAGCGATCCGCGAGCAGCCGGAGCAGGCCCGTCTGGCCCTGACCCTGGCCGCCGCCGAGAGCGAGCGCTTCGTCCGGCAGGGCACCGGCAACGACGAGGCCGGCGCGGCCAGCGCCGACGTGGTGAGCCTGACCTGCCCGGTCGCCGCCGGTGAATGCGCG

GGCCCGGCGGACAGCGGCGACGCCCTGCTGGAGCGCAACTATCCCACTGGCGCGGAGTTCCTCGGCGACGGCGGCGACGTCAGCTTCAGCACCCGCGGCACGCAGAACTGGACGGTGGAGCGGCTGCTCCAGGCGCACCGCCAACTGGAGGAGCGCGGCTATGTGTTCGTCGGCTACCACGGCACCTTCCTCGAAGCGGCGCAAAGCATCGTCTTCGGCGGGGTGCGCGCGCGCAGCCAGGACCTCGACGCGATCAGGCGCGGTTTCTATATCGCCGGCGATCCGGCGCTGGCCTACGGCTACGCCCAGGACCAGGAACCCGACGCGCGCGGCCGGATCCGCAACGGTGCCCTGCTGCGTGTCTATGTGCCGCGCTGGAGTCTGCCGGGCTTCTACCGCACCGGCCTGACCCTGGCCGCGCCGGAGGCGGCGGGCGAGGTCGAACGGCTGATCGGCCATCCGCTGCCGCTGCGCCTGGACGCCATCACCGGCCCCGAGGAGGAAGGCGGGCGCCTGGAGACCATTCTCGGCTGGCCGCTGGCCGAGCGCACCGTGGTGATTCCCTCGGCGATCCCCACCGACCCGCGCAACGTCGGCGGCGACCTCGACCCGTCCAGCATCCCCGACAAGGAACAGGCAATCAGCGCCCTGCCGGACTACGCCAGCCAGCCCGGCAAACCGCCGCGCGAGGACCTGAAG

**Genewiz**

GCCGAAGAGGCCTTTGATTTATGGAATGAATGCGCCAAGGCTTGTGTTCTGGACTTAAAAGATGGCGTGCGTAGCAGCCGCATGAGCGTGGATCCGGCCATTGCCGATACCAATGGCCAAGGTGTGCTGCATCACAGCATGGTGCTGGAGGGTGGCAACGATGCACTGAAACTGGCCTTTGACAACGCACTGAGCATCACCAGCGACGGTTTAACCATTCGTTTAGAAGGCGGCGTGGAGCCGAACAAAAAAGTTCGCTACAGTTATACCCGCCAAGCTCGCGGCAGCTGGTCTTTAAATTGGCTGGTTCCGATCGGCCACGAGAAACCGAGTAATATTAAAGTGTTTATTCATGAACTGAATGCTGGTAACCAACTGAGCCACATGAGCCCGATCTATACCATCGAGATGGGTGACGAACTGCTGGCCAAACTGGCCCGCGATGCCACCTTTTTTGTGCGCGCCCATGAGAGCAACGAGATGCAGCCTACTTTAGCCATCAGCCATGCTGGTGTGAGTGTGGTTATGGCACAAGCTCAGCCGCGCCGTGAGAAACGTTGGAGTGAGTGGGCAAGTGGCAAAGTGCTGTGTCTGCTGGACCCGCTGGATGGTGTGTACAATTATTTAGCCCAGCAGCGCTGCAATCTGGATGACACTTGGGAGGGCAAGATTTATCGCGTGCTGGCCGGTAATCCCGCTAAGCATGATTTAGATATCAAACCGACAGTGATTAGTCACCGTTTACACTTTCCGGAGGGCGGTAGTCTGGCAGCACTGACAGCCCATCAAGCTTGTCATCTGCCGTTAGAGACCTTCACACGCCATCGTCAGCCTCGCGGTTGGGAGCAGCTGGAGCAGTGCGGCTATCCCGTTCAGCGTCTGGTTGCTTTATATTTAGCCGCACGTCTGAGCTGGAATCAAGTTGACCAAGTTATTCGCAACGCCTTAGCAAGCCCGGGCAGTGGTGGTGATCTGGGCGAGGCAATCCGCGAACAGCCGGAGCAAGCTCGTCTGGCATTAACTTTAGCCGCAGCCGAAAGTGAACGTTTCGTTCGTCAAGGTACCGGCAACGATGAAGCTGGTGCAGCAAGCGCCGACGTGGTGAGTCTGACTTGTCCCGTTGCCGCTGGTGAATGCGCTGGTCCGGCAGATAGCGGCGATGCTTTATTAGAACGCAATTATCCGACCGGCGCAGAATTTCTGGGCGATGGCGGCGATGTTAGCTTTAGCACCCGTGGCACCCAGAATTGGACCGTTGAACGTCTGCTGCAAGCTCACCGCCAGCTGGAAGAACGCGGCTATGTGTTTGTGGGCTATCACGGCACCTTTCTGGAGGCCGCACAGAGTATTGTGTTCGGCGGTGTGCGCGCCCGTAGCCAAGACTTAGATGCCATTCGCCGCGGCTTCTATATTGCCGGCGACCCGGCCTTAGCATATGGCTATGCACAAGATCAAGAACCGGATGCCCGTGGCCGTATCCGCAATGGTGCACTGCTGCGTGTGTATGTGCCTCGCTGGTCTTTACCGGGTTTTTATCGCACCGGCTTAACTTTAGCAGCCCCCGAAGCAGCCGGCGAAGTGGAACGTTTAATTGGCCATCCGCTGCCGTTACGTCTGGATGCTATTACCGGCCCCGAAGAAGAAGGTGGCCGTTTAGAGACCATTTTAGGTTGGCCGCTGGCAGAACGCACCGTTGTTATTCCGAGTGCCATTCCTACCGATCCTCGTAATGTGGGTGGCGATCTGGATCCGAGTAGCATTCCGGATAAAGAGCAAGCTATCAGTGCCTTACCGGATTACGCAAGTCAGCCGGGTAAACCGCCGCGTGAGGATTTAAAA

**ThermoFisher**

GCCGAAGAGGCATTTGATCTGTGGAATGAATGTGCAAAAGCATGTGTTCTGGATCTGAAAGATGGTGTTCGTAGCAGCCGTATGAGCGTTGATCCGGCAATTGCAGATACCAATGGTCAGGGTGTTCTGCATCATTCAATGGTTCTGGAAGGTGGTAATGATGCACTGAAACTGGCCTTTGATAATGCACTGAGCATTACCAGTGATGGTCTGACCATTCGTCTGGAAGGCGGTGTTGAACCGAACAAAAAAGTTCGTTATAGTTATACCCGTCAGGCACGTGGTAGCTGGTCACTGAATTGGCTGGTTCCGATTGGTCATGAAAAACCGAGCAACATCAAAGTGTTTATCCATGAACTGAATGCCGGTAATCAGCTGAGCCATATGAGCCCGATTTATACCATTGAAATGGGTGATGAACTGCTGGCAAAACTGGCACGTGATGCAACCTTTTTTGTGCGTGCACATGAAAGCAATGAAATGCAGCCGACACTGGCAATTAGCCATGCCGGTGTTAGCGTTGTTATGGCACAGGCACAGCCTCGTCGTGAAAAACGTTGGAGCGAATGGGCAAGCGGTAAAGTTCTGTGTCTGCTGGATCCGCTGGATGGTGTTTATAACTATCTGGCACAGCAGCGTTGTAATCTGGATGATACCTGGGAAGGTAAAATCTATCGTGTTCTGGCAGGTAATCCGGCAAAACATGATCTGGATATTAAACCGACCGTTATTAGCCATCGTCTGCATTTTCCGGAAGGTGGCAGCCTGGCAGCACTGACCGCACATCAGGCATGTCATCTGCCGCTGGAAACCTTTACACGTCATCGTCAGCCTCGTGGTTGGGAACAGCTGGAACAGTGTGGTTATCCGGTTCAGCGTCTGGTTGCACTGTATCTGGCAGCCCGTCTGAGCTGGAATCAGGTTGATCAGGTTATTCGTAATGCCCTGGCAAGCCCTGGTAGCGGTGGTGATCTGGGTGAAGCAATTCGTGAACAGCCTGAACAGGCACGTCTGGCACTGACCCTGGCAGCCGCAGAAAGCGAACGTTTTGTTCGTCAAGGCACCGGCAATGATGAAGCCGGTGCAGCAAGCGCAGATGTTGTTAGCCTGACCTGTCCGGTTGCAGCCGGTGAATGTGCCGGTCCGGCAGATAGCGGTGATGCCCTGCTGGAACGTAATTATCCGACCGGTGCAGAATTTTTAGGTGATGGTGGTGATGTTAGCTTTAGCACCCGTGGCACCCAGAATTGGACCGTTGAACGTCTGCTGCAGGCACACCGTCAGCTGGAAGAACGTGGTTATGTTTTTGTTGGTTATCATGGCACCTTTCTGGAAGCAGCACAGAGCATTGTTTTTGGTGGTGTGCGTGCCCGTAGCCAGGATCTGGATGCAATTCGTCGTGGTTTCTATATTGCAGGCGATCCGGCACTGGCATATGGTTATGCACAGGATCAAGAACCGGATGCACGTGGTCGTATTCGCAATGGTGCACTGCTGCGTGTTTATGTTCCGCGTTGGAGCCTGCCTGGTTTTTATCGTACCGGTCTGACCTTAGCAGCACCGGAAGCAGCGGGTGAAGTGGAACGTCTGATTGGCCATCCGCTGCCGCTGCGTCTGGATGCCATTACCGGTCCGGAAGAAGAAGGCGGTCGCCTGGAAACCATTTTAGGTTGGCCTCTGGCAGAACGTACCGTTGTTATTCCGAGCGCAATTCCGACCGATCCGCGTAATGTTGGTGGCGATCTGGATCCGAGCAGCATTCCGGATAAAGAACAGGCCATTAGCGCACTGCCTGATTATGCAAGCCAGCCTGGTAAACCGCCTCGTGAAGATCTGAAA

**BiLSTM-CRF（a）**

GCGGAAGAAGCGTTTGATCTGTGGAACGAATGTGCGAAAGCCTGTGTGCTGGATCTGAAAGATGGCGTGCGTTCCAGCCGTATGAGCGTCGATCCGGCGATTGCCGATACCAACGGTCAGGGCGTGCTGCATCATTCGATGGTGCTGGAAGGCGGTAACGATGCGCTGAAACTGGCGTTTGATAACGCGCTGAGCATTACCAGCGATGGTCTGACCATTCGTCTGGAAGGCGGCGTGGAACCGAACAAAAAAGTGCGTTATAGCTATACCCGTCAGGCGCGTGGTAGCTGGTCGCTGAACTGGCTGGTGCCGATTGGTCATGAAAAACCGAGCAATATTAAAGTGTTTATTCATGAACTGAACGCCGGTAACCAGCTGAGCCATATGAGCCCGATTTATACCATTGAAATGGGCGATGAACTGCTGGCGAAACTGGCGCGTGATGCGACCTTCTTTGTCCGTGCGCATGAAAGCAACGAAATGCAGCCGACGCTGGCGATTAGCCATGCGGGCGTCAGCGTGGTGATGGCGCAGGCGCAGCCGCGTCGTGAAAAACGCTGGAGCGAATGGGCCAGCGGTAAAGTGCTGTGCCTGCTGGATCCGCTGGATGGCGTCTATAACTATCTGGCGCAGCAACGTTGTAACCTGGATGATACCTGGGAAGGTAAAATTTATCGCGTGCTGGCGGGTAACCCGGCGAAACATGATCTGGATATTAAACCGACCGTGATCAGCCATCGTCTGCATTTCCCGGAAGGCGGTTCGCTGGCGGCGCTGACCGCGCATCAGGCGTGCCATCTGCCGCTGGAAACCTTTACCCGCCATCGTCAGCCGCGCGGCTGGGAACAACTGGAACAATGCGGTTATCCGGTGCAGCGTCTGGTGGCGCTGTATCTGGCGGCGCGTCTGAGCTGGAACCAGGTGGATCAGGTGATTCGTAACGCGCTGGCGTCGCCGGGCAGCGGCGGCGATCTGGGTGAAGCGATTCGTGAACAACCGGAACAGGCGCGTCTGGCGCTGACGCTGGCGGCGGCGGAAAGCGAACGTTTTGTCCGTCAGGGCACCGGTAACGATGAAGCGGGTGCGGCGAGCGCCGATGTGGTCAGCCTGACCTGCCCGGTGGCGGCGGGTGAATGTGCGGGTCCGGCGGATAGCGGCGATGCGCTGCTGGAACGTAACTATCCGACCGGCGCGGAATTTCTGGGCGATGGCGGCGATGTCAGCTTCTCCACCCGCGGTACGCAAAACTGGACCGTTGAACGTCTGCTGCAGGCGCATCGTCAGCTGGAAGAGCGTGGTTATGTCTTTGTCGGTTATCACGGCACCTTCCTGGAAGCGGCGCAAAGCATTGTCTTTGGCGGCGTGCGTGCGCGTAGCCAGGATCTGGATGCGATTCGTCGCGGTTTTTATATTGCCGGTGATCCGGCGCTGGCGTATGGTTATGCGCAGGATCAGGAACCGGATGCGCGTGGTCGTATTCGTAACGGCGCGCTGCTGCGCGTCTATGTGCCGCGCTGGTCGCTGCCGGGTTTTTATCGCACCGGTCTGACGCTGGCGGCGCCGGAAGCGGCGGGTGAAGTGGAACGTCTGATTGGTCATCCGCTGCCGCTGCGTCTGGATGCGATTACCGGTCCGGAAGAAGAAGGCGGTCGTCTGGAAACCATTCTGGGCTGGCCGCTGGCGGAACGTACCGTGGTGATTCCGAGCGCCATTCCGACCGATCCGCGTAACGTCGGCGGCGATCTGGATCCGTCCAGCATTCCGGATAAAGAGCAGGCGATTAGCGCGCTGCCGGATTATGCCAGCCAGCCGGGTAAACCGCCGCGTGAAGATCTGAAA

**BiLSTM-CRF(b):**

GCGGAAGAAGCGTTTGATCTGTGGAACGAATGCGCGAAAGCGTGCGTGCTGGATCTGAAAGATGGCGTGCGTAGCAGCCGCATGAGCGTGGATCCGGCTATTGCCGATACCAACGGTCAGGGCGTGCTGCATCATTCCATGGTGCTGGAAGGCGGTAACGATGCGCTGAAACTGGCGTTTGATAACGCGCTGAGCATTACCAGCGATGGCCTGACCATTCGCCTGGAAGGCGGCGTGGAACCGAACAAAAAAGTGCGTTATTCCTATACCCGCCAGGCGCGTGGCAGCTGGAGCCTGAACTGGCTGGTGCCGATTGGTCATGAAAAACCGAGCAACATCAAAGTGTTTATTCATGAACTGAACGCCGGTAACCAGCTGAGCCACATGAGCCCGATTTATACCATTGAAATGGGCGATGAACTGCTGGCGAAACTGGCGCGTGATGCGACGTTTTTTGTTCGTGCGCATGAAAGCAACGAAATGCAGCCGACGCTGGCGATTTCCCATGCCGGCGTGAGCGTGGTGATGGCGCAGGCGCAGCCGCGCCGTGAAAAACGCTGGAGCGAATGGGCGAGCGGTAAAGTGCTGTGCCTGCTGGATCCGCTGGATGGCGTTTATAACTATCTGGCGCAGCAGCGCTGCAACCTGGATGATACCTGGGAAGGTAAAATTTATCGCGTGCTGGCGGGCAACCCGGCAAAACATGATCTGGATATTAAACCGACCGTTATTTCCCATCGCCTGCATTTTCCGGAAGGCGGCAGCCTGGCGGCGCTGACCGCCCATCAGGCGTGCCATCTGCCGCTGGAAACCTTTACCCGCCATCGCCAGCCGCGTGGCTGGGAACAGCTGGAACAGTGCGGTTATCCGGTGCAGCGTCTGGTGGCGCTGTATCTGGCGGCGCGTCTGAGCTGGAACCAGGTGGATCAGGTGATTCGCAACGCGCTGGCGAGCCCGGGTAGCGGCGGCGATCTGGGCGAAGCGATTCGTGAACAGCCGGAACAGGCGCGTCTGGCGCTGACGCTGGCGGCGGCGGAAAGCGAACGTTTTGTTCGTCAGGGCACCGGCAACGATGAAGCGGGCGCGGCGAGCGCGGATGTGGTGAGCCTGACCTGCCCGGTGGCGGCGGGCGAATGCGCGGGTCCGGCCGATAGCGGCGATGCGCTGCTGGAACGTAACTACCCGACCGGCGCGGAATTTCTGGGCGATGGCGGCGATGTTTCGTTTAGCACCCGCGGTACGCAGAACTGGACCGTGGAACGTCTGCTGCAGGCGCATCGCCAGCTGGAAGAACGCGGTTATGTCTTTGTCGGTTATCACGGTACGTTTCTGGAAGCGGCGCAGAGCATTGTCTTTGGCGGCGTGCGTGCGCGTAGCCAGGATCTGGATGCGATTCGCCGCGGTTTTTATATTGCCGGCGATCCGGCGCTGGCGTATGGTTATGCCCAGGATCAGGAACCGGATGCGCGTGGCCGTATTCGCAACGGCGCGCTGCTGCGCGTTTATGTTCCGCGCTGGAGCCTGCCGGGTTTTTATCGCACCGGTCTGACGCTGGCGGCGCCGGAAGCGGCGGGCGAAGTGGAACGTCTGATTGGTCATCCGCTGCCGCTGCGTCTGGATGCGATTACCGGTCCGGAAGAAGAAGGCGGTCGTCTGGAAACCATTCTGGGCTGGCCGCTGGCGGAACGTACCGTGGTGATTCCGAGCGCGATTCCGACCGATCCGCGTAACGTCGGCGGCGATCTGGATCCGAGCAGCATTCCGGATAAAGAACAGGCGATTTCCGCGCTGCCGGATTATGCCAGCCAGCCGGGTAAACCGCCGCGTGAAGATCTGAAA

**Mmpl3:**

**Original**

GTGTTCGCCTGGTGGGGTCGAACTGTGTACCGCTACCGGTTCATCGTAATCGGGGTCATGGTCGCTCTATGCCTCGGCGGCGGCGTTTTCGGGCTGAGCCTCGGCAAGCACGTCACGCAGAGCGGCTTCTACGACGACGGCAGCCAATCGGTGCAAGCATCGGTGCTGGGCGACCAGGTCTACGGCCGAGACCGAAGCGGTCACATCGTCGCGATCTTCCAAGCCCCAGCCGGCAAGACCGTTGACGACCCGGCCTGGTCAAAGAAGGTCGTCGACGAGCTCAACCGGTTCCAGCAGGATCACCCCGACCAGGTCTTGGGATGGGCCGGCTACCTGAGAGCGAGTCAGGCGACCGGCATGGCCACCGCCGACAAGAAGTACACCTTCGTTTCCATCCCGCTCAAGGGTGATGACGACGACACCATCCTCAACAACTACAAGGCCATCGCACCCGACCTGCAGCGGCTCGACGGAGGCACGGTGAAGCTCGCCGGGCTGCAACCGGTGGCCGAGGCGTTGACCGGCACCATCGCCACCGACCAACGGCGAATGGAAGTGCTGGCGCTGCCGTTGGTGGCGGTGGTGTTGTTCTTCGTGTTCGGCGGCGTGATCGCCGCCGGCCTACCGGTGATGGTCGGAGGGCTGTGCATCGCCGGCGCGCTGGGCATCATGCGGTTCCTCGCGATCTTCGGTCCCGTGCACTATTTCGCCCAGCCCGTGGTGTCGCTGATCGGTCTGGGGATCGCCATCGACTACGGGTTGTTCATCGTGAGCCGGTTCCGCGAAGAGATCGCCGAAGGCTACGACACCGAGACGGCAGTACGGCGCACGGTGATCACCGCCGGACGCACGGTGACGTTCTCGGCGGTGTTGATCGTCGCGTCGGCGATCGGTCTGCTGCTCTTCCCGCAGGGTTTCCTGAAGTCGCTGACCTACGCCACGATCGCATCGGTGATGCTGTCGGCCATCCTGTCTATCACCGTGTTGCCGGCCTGTCTGGGGATCCTGGGCAAACACGTCGACGCGCTCGGCGTGCGGACCCTGTTCCGGGTGCCCTTCCTGGCGAACTGGAAGATTTCGGCCGCCTACCTGAACTGGCTCGCCGACCGCCTGCAGCGGACCAAGACCCGCGAAGAGGTCGAAGCCGGCTTCTGGGGCAAGCTGGTTAACCGGGTGATGAAGCGCCCAGTGCTGTTCGCCGCACCGATCGTCATCATCATGATTTTGCTGATTATCCCGGTGGGCAAGCTGTCATTGGGCGGGATCAGCGAGAAGTACTTGCCGCCGACCAATTCGGTGCGCCAGGCGCAGGAGGAGTTCGACAAACTCTTCCCCGGATACCGCACCAATCCGCTGACACTGGTGATCCAGACCAGCAACCATCAACCGGTCACCGACGCGCAGATCGCTGACATCCGCAGCAAGGCGATGGCGATCGGCGGATTCATCGAGCCGGACAACGATCCGGCGAATATGTGGCAAGAGCGTGCCTACGCGGTAGGCGCATCTAAAGATCCATCGGTGCGCGTCCTGCAGAACGGGTTGATCAACCCGGCTGACGCGTCGAAGAAGCTCACCGAGCTGCGCGCGATCACCCCGCCCAAAGGAATCACGGTCTTGGTCGGTGGAACTCCCGCCCTGGAGCTGGATTCAATCCACGGCCTGTTCGCGAAGATGCCGCTGATGGTGGTCATCCTGCTGACCACCACGATCGTCTTGATGTTCTTGGCGTTCGGCTCGGTGGTGCTGCCAATCAAGGCGACGCTGATGAGCGCTCTGACGCTCGGGTCCACCATGGGCATCCTGACGTGGATATTCGTCGACGGACACTTTTCGAAGTGGCTGAATTTCACGCCGACCCCGCTGACAGCGCCGGTGATCGGGCTGATCATCGCGCTGGTCTTCGGCCTATCCACCGACTACGAGGTGTTCTTGGTGTCCCGGATGGTCGAGGCGCGAGAGCGCGGCATGTCGACCCAGGAGGCGATCCGGATCGGCACCGCAGCCACCGGACGCATCATTACCGCCGCGGCGCTGATTGTTGCCGTCGTCGCGGGCGCGTTCGTGTTCTCCGACCTGGTGATGATGAAGTATCTGGCCTTTGGACTGATGGCGGCGCTGCTGCTGGACGCGACCGTGGTGCGGATGTTTTTAGTGCCATCGGTGATGAAGCTGCTCGGCGATGACTGCTGGTGGGCACCGCGCTGGGCCAGACGCCTGCAGACCCGCATCGGGCTGGGCGAGATCCACCTGCCCGACGAGCGCAAGCGGCCCGTCAGCAACGGGCGTCCCGCACGTCCTCCGGTCACAGCTGGGCTGGTTGCGGCGCGCGCCGCTGGGGACCCGCGCCCACCGCACGATCCGACCCATCCGCTGGCGGAGTCACCTCGACCGGCCCGCTCGAGTCCAGCAAGCTCACCGGAGCTCACGCCTGCCCTGGAAGCAACTGCCGCGCCGGCGGCGCCGTCTGGGGCGAGCACCACACGGATGCAGATCGGGTCGTCGACGGAGCCGCCGACAACCCGCCTCGCGGCTGCCGGTCGGTCCGTGCAGTCGCCAGCATCCACGCCGCCACCAACCCCGACCCCGCCATCGGCCCCGTCTGCCGGTCAGACCCGGGCTATGCCGCTTGCGGCGAACCGCTCCACAGACGCAGCCGGTGACCCGGCCGAACCCACCGCGGCCCTGCCAATCATACGGTCGGACGGCGACGACTCAGAGGCAGCCACTGAGCAGCTGAATGCCCGCGGCACGAGCGATAAGACGCGTCAGCGCCGCCGCGGCGGCGGCGCCCTGTCCGCCCAGGATCTGCTTCGCCGCGAAGGACGCCTTTAA

**Genewiz**

GTTTTCGCATGGTGGGGCCGTACAGTGTATCGCTATCGCTTTATCGTTATTGGTGTGATGGTGGCTTTATGTTTAGGTGGCGGTGTGTTCGGTCTGAGCTTAGGCAAACACGTGACCCAGAGTGGTTTCTACGACGACGGTAGCCAGAGTGTGCAAGCTAGCGTTCTGGGTGACCAAGTTTACGGTCGCGACCGCAGTGGTCATATCGTTGCCATCTTTCAAGCTCCGGCTGGTAAGACCGTGGACGATCCGGCATGGAGCAAAAAAGTTGTTGATGAGCTGAATCGCTTCCAGCAAGATCACCCGGACCAAGTTCTGGGCTGGGCTGGTTATTTACGCGCCAGCCAAGCTACTGGTATGGCCACAGCCGACAAAAAGTATACCTTCGTTAGCATTCCGCTGAAAGGTGACGATGATGATACCATTTTAAATAACTACAAAGCAATTGCCCCCGATCTGCAACGTCTGGATGGTGGTACCGTGAAACTGGCTGGTCTGCAGCCGGTGGCCGAAGCACTGACCGGTACCATCGCCACCGATCAACGTCGCATGGAAGTTTTAGCACTGCCGCTGGTGGCAGTGGTGCTGTTCTTTGTGTTCGGCGGTGTTATCGCCGCCGGCTTACCGGTGATGGTTGGCGGTCTGTGTATTGCTGGTGCACTGGGCATTATGCGCTTTTTAGCAATTTTCGGCCCGGTTCACTACTTTGCCCAACCGGTGGTGTCTTTAATTGGTTTAGGTATTGCAATCGACTATGGTCTGTTCATTGTGAGCCGCTTCCGCGAGGAAATTGCCGAAGGCTATGACACCGAGACAGCCGTGCGCCGCACAGTGATCACCGCCGGTCGTACCGTTACATTTAGCGCCGTGCTGATCGTGGCAAGTGCAATCGGTTTACTGTTATTCCCTCAAGGTTTTTTAAAGAGTCTGACCTACGCAACCATCGCCAGCGTTATGCTGAGCGCCATTCTGTCTATCACCGTTCTGCCGGCATGTTTAGGCATTCTGGGCAAACATGTGGATGCTTTAGGTGTGCGTACTTTATTCCGTGTGCCGTTTTTAGCAAATTGGAAGATCAGCGCAGCCTATTTAAATTGGCTGGCAGATCGTTTACAACGTACCAAGACCCGTGAAGAAGTTGAAGCCGGCTTTTGGGGCAAGTTAGTGAACCGTGTGATGAAGCGCCCGGTTCTGTTTGCCGCCCCTATCGTTATCATTATGATTTTACTGATTATTCCCGTTGGCAAGCTGTCTTTAGGTGGCATTAGCGAGAAATATTTACCCCCTACCAATAGCGTGCGTCAAGCTCAAGAAGAGTTTGACAAGCTGTTTCCCGGTTACCGTACAAACCCGCTGACTTTAGTGATTCAGACCAGCAACCATCAGCCGGTTACAGATGCCCAGATCGCAGATATCCGCAGCAAAGCAATGGCAATCGGTGGCTTCATTGAGCCCGATAATGACCCGGCCAACATGTGGCAAGAACGCGCATACGCAGTGGGTGCCAGTAAAGATCCGAGCGTTCGTGTTCTGCAGAACGGTTTAATTAATCCGGCCGACGCAAGCAAGAAACTGACAGAGCTGCGTGCCATTACACCGCCGAAAGGCATCACTGTTCTGGTTGGCGGTACTCCGGCTTTAGAACTGGACAGCATCCATGGTTTATTCGCAAAGATGCCGCTGATGGTTGTGATCTTACTGACCACCACCATCGTTCTGATGTTTTTAGCCTTCGGCAGTGTGGTGCTGCCGATTAAAGCCACTTTAATGAGTGCCTTAACACTGGGTAGCACCATGGGTATTCTGACATGGATCTTTGTGGACGGCCACTTCAGCAAGTGGCTGAACTTTACACCGACCCCTCTGACCGCACCGGTGATTGGCTTAATTATCGCTTTAGTGTTTGGTTTAAGCACCGATTATGAGGTGTTTCTGGTGAGCCGCATGGTGGAGGCCCGTGAACGTGGCATGAGTACACAAGAAGCCATTCGCATTGGCACCGCAGCAACCGGTCGCATCATTACAGCCGCCGCTTTAATTGTTGCCGTTGTGGCCGGCGCCTTTGTTTTTAGTGATTTAGTTATGATGAAGTATCTGGCTTTCGGTTTAATGGCAGCACTGCTGCTGGATGCAACCGTTGTGCGTATGTTTTTAGTGCCTAGCGTGATGAAACTGCTGGGCGATGACTGCTGGTGGGCCCCTCGTTGGGCACGTCGTTTACAAACCCGCATCGGCTTAGGTGAGATCCACTTACCGGATGAGCGTAAACGCCCCGTTAGCAACGGTCGTCCGGCACGCCCGCCCGTTACCGCTGGTTTAGTTGCAGCCCGTGCAGCTGGTGATCCGCGTCCCCCTCACGATCCGACACATCCGCTGGCAGAAAGCCCTCGTCCCGCTCGTAGCAGCCCGGCCAGTAGTCCCGAATTAACACCGGCTCTGGAAGCCACCGCAGCACCCGCTGCCCCTAGTGGTGCAAGCACCACACGTATGCAGATCGGCAGCAGCACAGAACCTCCGACCACCCGCTTAGCAGCAGCTGGTCGCAGTGTTCAGAGCCCCGCTAGTACACCGCCTCCGACACCTACACCTCCGAGCGCACCGAGTGCTGGTCAGACACGTGCAATGCCGCTGGCAGCAAACCGTAGCACAGATGCAGCTGGTGATCCGGCAGAACCGACCGCAGCACTGCCGATCATCCGCAGCGATGGCGACGACAGTGAGGCCGCAACCGAACAGCTGAATGCCCGCGGTACCAGCGACAAAACACGCCAACGCCGTCGTGGTGGTGGCGCCTTAAGCGCACAAGATCTGCTGCGCCGCGAGGGCCGTCTGTAA

**ThermoFisher**

GTTTTTGCATGGTGGGGTCGTACCGTTTATCGTTATCGTTTTATTGTGATTGGTGTTATGGTTGCCCTGTGTTTAGGTGGTGGTGTTTTTGGTCTGAGCCTGGGTAAACATGTGACCCAGAGCGGTTTTTATGATGATGGTAGCCAGAGCGTTCAGGCAAGCGTTCTGGGTGATCAGGTTTATGGTCGTGATCGTAGCGGTCATATTGTTGCAATTTTTCAGGCACCGGCAGGTAAAACCGTTGATGATCCGGCATGGTCAAAAAAAGTTGTGGATGAACTGAATCGCTTTCAGCAGGATCATCCGGATCAGGTGTTAGGTTGGGCAGGTTATCTGCGTGCAAGCCAGGCAACCGGTATGGCAACCGCAGATAAAAAGTATACCTTTGTTAGCATTCCGCTGAAAGGTGATGATGATGACACCATTCTGAACAACTATAAAGCAATTGCACCGGATCTGCAGCGTCTGGATGGTGGCACCGTTAAACTGGCAGGTCTGCAGCCGGTTGCCGAAGCACTGACCGGCACCATTGCAACCGATCAGCGTCGTATGGAAGTTCTGGCACTGCCGCTGGTTGCAGTTGTTCTGTTTTTTGTTTTTGGCGGTGTTATTGCAGCGGGTCTGCCGGTGATGGTTGGTGGTCTGTGTATTGCCGGTGCACTGGGTATTATGCGTTTTCTGGCAATTTTTGGTCCGGTGCATTATTTTGCACAGCCGGTGGTTAGCCTGATTGGTCTGGGCATTGCAATTGATTATGGCCTGTTTATTGTTAGCCGTTTTCGTGAAGAAATTGCCGAAGGTTATGATACCGAAACCGCAGTTCGTCGCACCGTTATTACCGCAGGCCGTACCGTTACCTTTAGCGCAGTTCTGATTGTTGCCAGCGCAATTGGTCTGCTGCTGTTTCCGCAGGGTTTTCTGAAAAGCCTGACCTATGCAACCATTGCCAGCGTTATGCTGAGCGCAATTCTGAGCATTACCGTTCTGCCTGCATGTCTGGGTATTCTGGGCAAACACGTTGATGCCCTGGGTGTTCGTACCCTGTTTCGTGTTCCGTTTCTGGCCAATTGGAAAATTAGCGCAGCATATCTGAATTGGCTGGCAGATCGTCTGCAGCGCACCAAAACACGTGAAGAAGTTGAAGCAGGTTTTTGGGGTAAACTGGTTAATCGTGTTATGAAACGTCCGGTTCTGTTTGCAGCACCGATTGTGATTATTATGATTCTGCTGATTATTCCGGTTGGCAAACTGAGCTTAGGTGGTATTAGCGAAAAATATCTGCCTCCGACCAATAGCGTTCGTCAGGCACAAGAAGAATTCGATAAACTGTTTCCGGGTTATCGTACCAATCCGCTGACACTGGTTATTCAGACCAGCAATCATCAGCCTGTTACCGATGCACAGATTGCAGATATTCGTAGCAAAGCAATGGCAATTGGTGGTTTTATTGAACCGGATAACGATCCGGCAAATATGTGGCAAGAACGTGCCTATGCAGTTGGTGCAAGCAAAGATCCGAGCGTTCGTGTGCTGCAGAATGGTCTGATTAATCCGGCAGATGCCAGCAAAAAACTGACCGAACTGCGTGCGATTACCCCTCCGAAAGGTATTACCGTGCTGGTTGGCGGTACACCGGCACTGGAACTGGATAGCATTCATGGTCTGTTTGCCAAAATGCCGCTGATGGTGGTTATTCTGTTAACCACCACCATTGTTCTGATGTTTCTGGCATTTGGTAGCGTTGTTCTGCCGATTAAAGCAACCCTGATGAGCGCACTGACCCTGGGTAGCACCATGGGCATTCTGACCTGGATTTTTGTTGATGGCCATTTTAGCAAATGGCTGAATTTTACCCCGACACCGCTGACCGCACCGGTTATTGGTCTGATCATTGCCCTGGTTTTTGGCCTGTCAACCGATTATGAAGTTTTTCTGGTTAGCCGTATGGTTGAAGCACGTGAACGTGGTATGAGCACCCAAGAAGCCATTCGTATTGGCACCGCAGCAACCGGTCGTATTATTACAGCAGCCGCACTGATTGTGGCAGTTGTTGCGGGTGCATTTGTTTTTAGCGATCTGGTCATGATGAAATATCTGGCCTTTGGCCTGATGGCAGCACTGCTGCTGGATGCCACCGTTGTGCGTATGTTTTTAGTTCCGAGCGTGATGAAACTGCTGGGTGATGACTGTTGGTGGGCACCGCGTTGGGCACGTCGCCTGCAGACCCGTATTGGCCTGGGTGAAATTCATCTGCCTGATGAACGTAAACGTCCTGTTAGCAATGGTCGTCCGGCACGTCCTCCGGTGACCGCAGGTCTGGTAGCAGCACGTGCAGCAGGCGATCCGCGTCCGCCTCATGATCCGACACATCCGCTGGCAGAATCACCGCGTCCTGCACGTAGCAGTCCGGCAAGCTCACCGGAACTGACCCCTGCACTGGAAGCAACAGCAGCTCCGGCAGCACCGTCAGGTGCCAGCACCACACGTATGCAGATTGGTAGCAGTACCGAACCGCCTACCACACGTCTGGCAGCCGCAGGTCGTAGCGTTCAGAGTCCGGCATCAACACCGCCTCCGACACCGACACCTCCGAGCGCACCTAGCGCAGGTCAGACCCGTGCTATGCCTCTGGCAGCAAATCGTAGCACCGATGCTGCGGGTGATCCTGCAGAACCGACAGCAGCCCTGCCGATCATTCGTAGTGATGGTGATGATAGCGAAGCAGCCACCGAACAGCTGAATGCACGTGGCACCAGCGATAAAACCCGTCAGCGTCGCCGTGGTGGTGGCGCACTGAGCGCACAAGATCTGCTGCGTCGTGAAGGTCGTCTGTAA

**BiLSTM-CRF（a）：**

GTGTTTGCCTGGTGGGGTCGTACCGTCTATCGTTATCGTTTTATTGTCATTGGCGTGATGGTGGCGCTGTGCCTGGGCGGCGGCGTGTTTGGTCTGTCGCTGGGTAAACATGTCACCCAAAGCGGTTTTTATGATGATGGCAGCCAGAGCGTGCAGGCCAGCGTGCTGGGCGATCAGGTTTATGGTCGCGATCGCAGCGGTCATATTGTGGCGATTTTCCAGGCGCCGGCGGGTAAAACCGTCGATGATCCGGCGTGGAGCAAAAAAGTGGTGGATGAACTGAACCGTTTTCAGCAGGATCATCCGGATCAGGTGCTGGGCTGGGCGGGTTATCTGCGCGCCAGCCAGGCGACCGGTATGGCGACGGCGGATAAAAAATATACCTTTGTCAGCATTCCGCTGAAAGGCGATGATGATGATACCATTCTGAACAACTATAAAGCGATTGCGCCGGATCTGCAACGTCTGGATGGCGGCACCGTGAAACTGGCGGGTCTGCAACCGGTGGCGGAAGCGCTGACCGGCACCATTGCCACCGATCAGCGTCGTATGGAAGTGCTGGCGCTGCCGCTGGTGGCGGTGGTGCTGTTTTTTGTCTTTGGCGGCGTGATTGCGGCGGGTCTGCCGGTGATGGTCGGCGGTCTGTGTATTGCCGGTGCGCTGGGCATTATGCGTTTTCTGGCGATTTTTGGTCCGGTGCATTATTTTGCGCAGCCGGTGGTCAGCCTGATTGGTCTGGGCATTGCCATTGATTACGGTCTGTTTATTGTCAGCCGTTTCCGTGAAGAAATTGCTGAAGGTTATGATACCGAAACCGCGGTGCGTCGTACCGTGATTACCGCGGGTCGTACCGTCACCTTCAGCGCGGTGCTGATTGTCGCCAGCGCCATTGGTCTGCTGCTGTTCCCGCAGGGCTTCCTGAAATCGCTGACCTATGCCACCATTGCCAGCGTGATGCTGAGCGCCATTCTGAGCATTACCGTGCTGCCGGCGTGCCTGGGCATTCTGGGTAAACATGTCGATGCGCTGGGCGTGCGTACGCTGTTCCGCGTGCCGTTCCTGGCGAACTGGAAAATCAGCGCGGCGTATCTGAACTGGCTGGCGGATCGTCTGCAACGTACCAAAACCCGTGAAGAAGTGGAAGCGGGTTTCTGGGGTAAACTGGTCAACCGCGTGATGAAACGTCCGGTGCTGTTTGCGGCGCCGATTGTCATTATTATGATTCTGCTGATTATTCCGGTCGGTAAACTGTCGCTGGGCGGTATCAGCGAAAAATATCTGCCGCCGACCAACAGCGTGCGTCAGGCGCAGGAAGAGTTTGATAAACTGTTCCCGGGTTATCGCACCAACCCGCTGACGCTGGTGATTCAGACCAGCAACCATCAGCCGGTGACCGATGCGCAAATTGCCGATATTCGCAGCAAAGCGATGGCGATTGGCGGTTTTATTGAACCGGATAACGATCCGGCGAATATGTGGCAGGAACGTGCGTATGCGGTGGGTGCCAGCAAAGATCCGAGCGTGCGCGTGCTGCAAAACGGTCTGATTAACCCGGCGGATGCCAGCAAAAAACTGACCGAACTGCGTGCGATTACCCCGCCGAAAGGCATTACCGTGCTGGTGGGCGGTACGCCGGCGCTGGAACTGGATAGCATTCATGGTCTGTTTGCGAAAATGCCGCTGATGGTGGTGATTCTGCTGACCACCACCATTGTGCTGATGTTCCTGGCGTTTGGCAGCGTGGTGCTGCCGATTAAAGCGACGCTGATGAGCGCGCTGACGCTGGGTTCCACCATGGGCATTCTGACCTGGATTTTTGTCGATGGTCATTTCAGCAAATGGCTGAACTTCACCCCGACGCCGCTGACCGCGCCGGTGATTGGTCTGATTATTGCGCTGGTGTTTGGTCTGAGCACCGATTATGAAGTGTTTCTGGTCAGCCGTATGGTGGAAGCGCGTGAACGCGGCATGAGCACCCAGGAAGCGATTCGTATTGGCACCGCGGCGACCGGTCGTATTATTACCGCGGCGGCGCTGATTGTGGCGGTGGTGGCGGGTGCGTTTGTCTTCAGCGATCTGGTGATGATGAAATATCTGGCGTTTGGTCTGATGGCGGCGCTGCTGCTGGATGCGACCGTGGTGCGTATGTTCCTGGTGCCGTCGGTGATGAAACTGCTGGGCGATGATTGCTGGTGGGCGCCGCGCTGGGCGCGTCGTCTGCAAACCCGCATTGGTCTGGGTGAAATTCATCTGCCGGATGAACGTAAACGTCCGGTCAGCAACGGTCGTCCGGCGCGTCCGCCGGTGACCGCGGGTCTGGTGGCGGCGCGTGCGGCGGGCGATCCGCGTCCGCCGCATGATCCGACCCATCCGCTGGCGGAAAGCCCGCGTCCGGCGCGTTCCAGCCCGGCATCCAGCCCGGAACTGACGCCGGCGCTGGAAGCGACCGCGGCGCCGGCGGCGCCGTCCGGTGCCAGCACCACCCGCATGCAGATTGGTTCCAGCACCGAACCGCCGACCACCCGTCTGGCGGCGGCGGGTCGCAGCGTGCAGTCGCCGGCATCGACGCCGCCGCCGACGCCGACGCCGCCGTCGGCGCCGAGCGCCGGTCAGACCCGTGCGATGCCGCTGGCGGCGAACCGCAGCACCGATGCGGCGGGCGATCCGGCAGAACCGACCGCGGCGCTGCCGATTATTCGCAGCGATGGCGATGACAGCGAAGCGGCGACCGAACAACTGAACGCGCGTGGCACCAGCGATAAAACCCGTCAGCGTCGTCGCGGCGGCGGCGCGCTGAGCGCGCAGGATCTGCTGCGTCGTGAAGGTCGTCTGTAA

**BiLSTM-CRF（b）：**

GTGTTTGCCTGGTGGGGTCGTACCGTTTATCGCTATCGCTTTATTGTTATTGGCGTGATGGTGGCGCTGTGCCTGGGCGGCGGCGTGTTTGGTCTGTCGCTGGGTAAACATGTTACCCAGAGCGGCTTTTATGATGATGGCAGCCAGAGCGTGCAGGCGAGCGTGCTGGGCGATCAGGTTTATGGCCGTGATCGCAGCGGTCATATTGTCGCGATTTTTCAGGCGCCGGCCGGTAAAACCGTGGATGATCCGGCCTGGAGCAAAAAAGTGGTGGATGAACTGAACCGTTTTCAGCAGGATCATCCGGATCAGGTGCTGGGCTGGGCGGGTTATCTGCGTGCGAGCCAGGCGACCGGCATGGCGACCGCGGATAAAAAATACACCTTTGTTTCCATTCCGCTGAAAGGCGATGATGATGATACCATTCTGAACAACTACAAAGCGATTGCCCCGGATCTGCAGCGTCTGGATGGCGGTACGGTGAAACTGGCGGGTCTGCAGCCGGTGGCGGAAGCGCTGACCGGCACCATTGCCACCGATCAGCGCCGCATGGAAGTGCTGGCGCTGCCGCTGGTGGCGGTGGTGCTGTTTTTTGTTTTTGGCGGCGTGATTGCCGCCGGTCTGCCGGTGATGGTCGGCGGTCTGTGCATTGCCGGTGCGCTGGGCATTATGCGTTTTCTGGCGATTTTTGGTCCGGTGCATTATTTTGCCCAGCCGGTGGTGAGCCTGATTGGTCTGGGCATTGCCATTGATTATGGTCTGTTTATTGTTAGCCGTTTTCGTGAAGAAATTGCCGAAGGCTATGATACCGAAACCGCGGTGCGTCGTACCGTTATTACCGCCGGTCGTACCGTTACCTTTTCCGCGGTGCTGATTGTCGCGAGCGCGATTGGTCTGCTGCTGTTTCCGCAGGGCTTTCTGAAAAGCCTGACCTATGCCACCATTGCCAGCGTGATGCTGAGCGCGATTCTGAGCATTACCGTGCTGCCGGCGTGCCTGGGCATTCTGGGTAAACATGTCGATGCGCTGGGCGTGCGTACGCTGTTTCGCGTGCCGTTTCTGGCGAACTGGAAAATTTCCGCGGCGTATCTGAACTGGCTGGCGGATCGTCTGCAGCGTACCAAAACCCGTGAAGAAGTGGAAGCGGGTTTTTGGGGTAAACTGGTGAACCGCGTGATGAAACGCCCGGTGCTGTTTGCCGCACCGATTGTTATTATTATGATTCTGCTGATTATTCCGGTCGGTAAACTGTCGCTGGGCGGTATTAGCGAAAAATATCTGCCGCCGACCAACAGCGTGCGTCAGGCGCAGGAAGAGTTTGATAAACTGTTTCCGGGTTATCGCACCAACCCGCTGACGCTGGTGATTCAGACCAGCAACCATCAGCCGGTTACCGATGCGCAGATTGCCGATATTCGCAGCAAAGCGATGGCGATTGGCGGTTTTATTGAACCGGATAACGATCCGGCAAACATGTGGCAGGAACGTGCGTATGCCGTCGGCGCGAGCAAAGATCCGAGCGTTCGCGTGCTGCAGAACGGTCTGATTAACCCGGCCGATGCGAGCAAAAAACTGACCGAACTGCGTGCGATTACCCCGCCGAAAGGCATTACCGTGCTGGTCGGCGGTACGCCGGCGCTGGAACTGGATAGCATTCACGGTCTGTTTGCCAAAATGCCGCTGATGGTGGTGATTCTGCTGACCACCACCATTGTGCTGATGTTTCTGGCGTTTGGTAGCGTGGTGCTGCCGATTAAAGCGACGCTGATGAGCGCGCTGACGCTGGGCAGCACCATGGGCATTCTGACCTGGATTTTTGTTGATGGCCATTTTAGCAAATGGCTGAACTTTACCCCGACGCCGCTGACCGCCCCGGTTATTGGTCTGATTATTGCCCTGGTGTTTGGTCTGAGCACCGATTATGAAGTGTTTCTGGTGAGCCGCATGGTGGAAGCGCGTGAACGTGGCATGAGCACCCAGGAAGCGATTCGCATTGGCACCGCGGCGACCGGTCGTATTATTACCGCGGCGGCGCTGATTGTCGCGGTGGTGGCGGGCGCGTTTGTGTTTTCCGATCTGGTGATGATGAAATATCTGGCGTTTGGTCTGATGGCGGCGCTGCTGCTGGATGCGACCGTGGTGCGTATGTTTCTGGTTCCGAGCGTGATGAAACTGCTGGGCGATGATTGCTGGTGGGCTCCGCGCTGGGCGCGTCGTCTGCAGACCCGTATTGGTCTGGGCGAAATTCATCTGCCGGATGAACGTAAACGCCCGGTGAGCAACGGCCGTCCGGCGCGTCCGCCGGTTACCGCCGGTCTGGTGGCGGCGCGTGCGGCGGGCGATCCGCGCCCGCCGCATGATCCGACCCATCCGCTGGCGGAAAGCCCGCGCCCGGCCCGCAGCAGCCCGGCCAGCAGCCCGGAACTGACCCCGGCGCTGGAAGCGACCGCGGCGCCGGCGGCGCCGAGCGGCGCGAGCACCACCCGCATGCAGATTGGCAGCAGCACCGAACCGCCGACCACCCGCCTGGCGGCGGCGGGCCGTAGCGTGCAGAGCCCGGCCAGCACGCCGCCGCCGACGCCGACGCCGCCGAGCGCCCCGAGCGCCGGTCAGACCCGTGCGATGCCGCTGGCGGCGAACCGCAGCACCGATGCGGCGGGCGATCCGGCTGAACCGACCGCGGCGCTGCCGATTATTCGCAGCGATGGCGATGATAGCGAAGCGGCGACCGAACAGCTGAACGCCCGCGGTACGAGCGATAAAACCCGCCAGCGCCGTCGTGGCGGCGGCGCGCTGAGCGCGCAGGATCTGCTGCGTCGTGAAGGCCGTCTGTAA

**HPDF：**

**Original**

GAGGGCCCGGCGCTGGGGCGCTCCTATTGGCGCCACCTGAGGCGTCTGGTGCTGGGTCCTCCCGAACCGCCGTTCTCGCACGTGTGCCAAGTCGGGGACCCGGTGCTGCGCGGCGTGGCGGCCCCGGTGGAGCGGGCGCAGCTAGGCGGGCCCGAGCTGCAGCGGCTGACGCAACGGCTGGTCCAGGTGATGCGGCGGCGGCGCTGCGTGGGCCTAAGCGCGCCGCAGCTGGGGGTGCCGCGGCAGGTGCTGGCGCTGGAGCTCCCCGAGGCGCTGTGTCGGGAGTGCCCGCCCCGCCAGCGCGCGCTCCGCCAAATGGAGCCCTTCCCCCTGCGCGTGTTCGTGAACCCCAGCCTGCGAGTGCTTGACAGCCGCCTGGTCACCTTTCCCGAGGGCTGCGAGAGCGTCGCCGGCTTCCTGGCCTGCGTGCCCCGCTTCCAGGCGGTGCAGATCTCAGGGCTGGACCCCAATGGAGAACAGGTGGTGTGGCAGGCGAGCGGGTGGGCAGCCCGCATCATCCAGCACGAGATGGACCACCTGCAGGGCTGCCTGTTTATTGACAAAATGGACAGCAGGACGTTCACAAACGTCTATTGGATGAAGGTGAATGACTAA

**Genewiz**

GAAGGTCCGGCTTTAGGCCGTAGCTATTGGCGTCATCTGCGTCGTTTAGTGCTGGGTCCGCCGGAACCGCCGTTTAGCCATGTGTGCCAAGTTGGTGATCCCGTTCTGCGTGGTGTGGCAGCCCCGGTTGAACGTGCACAACTGGGTGGTCCGGAACTGCAGCGTTTAACCCAGCGTCTGGTTCAAGTTATGCGTCGCCGCCGTTGTGTTGGTCTGAGCGCACCGCAGCTGGGTGTTCCGCGTCAAGTTCTGGCTTTAGAACTGCCCGAAGCACTGTGTCGCGAATGTCCGCCGCGTCAGCGTGCATTACGTCAGATGGAACCGTTTCCGCTGCGCGTGTTTGTTAATCCGTCTTTACGTGTGCTGGACAGCCGTTTAGTGACCTTTCCGGAAGGTTGCGAAAGCGTTGCCGGCTTTCTGGCATGCGTGCCGCGCTTTCAAGCTGTGCAGATCAGCGGTCTGGATCCGAATGGCGAACAAGTTGTTTGGCAAGCCAGCGGTTGGGCCGCACGCATTATCCAGCACGAGATGGATCATTTACAAGGTTGTTTATTCATCGATAAGATGGACAGCCGCACCTTCACCAATGTGTATTGGATGAAAGTGAATGATTAA

**ThermoFisher**

GAAGGTCCGGCACTGGGTCGTAGCTATTGGCGTCATCTGCGTCGTCTGGTTCTGGGTCCGCCTGAACCGCCTTTTAGCCATGTTTGTCAGGTTGGTGATCCGGTTCTGCGTGGTGTTGCAGCACCGGTTGAACGTGCACAGTTAGGTGGTCCGGAACTGCAGCGTCTGACCCAGCGTCTGGTGCAGGTTATGCGTCGTCGTCGTTGTGTTGGTCTGAGCGCACCGCAGCTGGGTGTTCCGCGTCAGGTTCTGGCACTGGAACTGCCGGAAGCACTGTGTCGTGAATGTCCGCCTCGTCAGCGTGCACTGCGTCAGATGGAACCGTTTCCGCTGCGTGTTTTTGTTAATCCGAGCCTGCGTGTTCTGGATAGCCGTCTGGTTACCTTTCCGGAAGGTTGTGAAAGCGTTGCAGGTTTTCTGGCATGCGTTCCGCGTTTTCAGGCAGTTCAGATTAGCGGTCTGGATCCGAATGGTGAACAGGTTGTTTGGCAGGCAAGCGGTTGGGCAGCACGTATTATTCAGCATGAAATGGATCATCTGCAGGGCTGTCTGTTCATTGATAAAATGGATAGTCGCACCTTCACCAACGTGTATTGGATGAAAGTGAACGACTAA

**BiLSTM-CRF（a）**

GAAGGTCCGGCGCTGGGTCGCAGCTACTGGCGTCATCTGCGTCGTCTGGTGCTGGGTCCGCCGGAACCGCCGTTCAGCCATGTCTGCCAGGTCGGCGATCCGGTGCTGCGCGGCGTGGCGGCGCCGGTGGAACGTGCGCAACTGGGCGGTCCGGAACTGCAACGTCTGACCCAGCGTCTGGTGCAGGTGATGCGTCGTCGTCGCTGCGTCGGTCTGAGCGCGCCGCAACTGGGCGTGCCGCGTCAGGTGCTGGCGCTGGAACTGCCGGAAGCGCTGTGCCGTGAATGCCCGCCGCGTCAGCGTGCGCTGCGTCAGATGGAACCGTTCCCGCTGCGCGTGTTTGTCAACCCGTCGCTGCGCGTGCTGGATAGCCGTCTGGTGACCTTCCCGGAAGGCTGTGAAAGCGTGGCGGGTTTTCTGGCGTGTGTGCCGCGTTTTCAGGCGGTGCAGATCAGCGGTCTGGATCCGAACGGTGAACAGGTGGTCTGGCAGGCCAGCGGCTGGGCGGCGCGTATTATTCAGCATGAAATGGATCATCTGCAGGGCTGCCTGTTTATTGATAAAATGGATAGCCGTACCTTTACCAACGTCTATTGGATGAAAGTCAACGATTAA

**BiLSTM-CRF（b）**

GAAGGTCCGGCGCTGGGCCGTAGCTATTGGCGTCATCTGCGTCGTCTGGTGCTGGGTCCGCCGGAACCGCCGTTTTCTCATGTGTGCCAGGTCGGCGATCCGGTGCTGCGTGGCGTGGCGGCGCCGGTTGAACGTGCGCAGCTGGGCGGTCCGGAACTGCAGCGTCTGACCCAGCGTCTGGTGCAGGTGATGCGTCGTCGTCGTTGCGTCGGTCTGAGCGCCCCGCAGCTGGGCGTTCCGCGCCAGGTGCTGGCGCTGGAACTGCCGGAAGCGCTGTGCCGTGAATGCCCGCCGCGCCAGCGTGCGCTGCGTCAGATGGAACCGTTTCCGCTGCGCGTGTTTGTTAACCCGAGCCTGCGCGTGCTGGATAGCCGTCTGGTGACCTTTCCGGAAGGCTGCGAAAGCGTGGCGGGTTTTCTGGCGTGCGTGCCGCGTTTTCAGGCGGTGCAGATTTCCGGCCTGGATCCGAACGGCGAACAGGTGGTCTGGCAGGCGAGCGGCTGGGCGGCGCGTATTATTCAGCATGAAATGGATCATCTGCAGGGCTGCCTGTTTATTGATAAAATGGATAGCCGTACCTTTACCAACGTTTATTGGATGAAAGTGAACGATTAA
